# Patterns of Fitness and Gene Expression Epistasis Generated by Beneficial Mutations in the *rho* and *rpoB* Genes of *Escherichia coli* during High-Temperature Adaptation

**DOI:** 10.1093/molbev/msae187

**Published:** 2024-09-05

**Authors:** Andrea González-González, Tiffany N Batarseh, Alejandra Rodríguez-Verdugo, Brandon S Gaut

**Affiliations:** Department of Ecology and Evolutionary Biology, University of California, Irvine, Irvine, CA 92697, USA; Department of Biology, University of Florida, Gainesville, FL, USA; Department of Ecology and Evolutionary Biology, University of California, Irvine, Irvine, CA 92697, USA; Department of Integrative Biology, UC Berkeley, Berkeley, CA, USA; Department of Ecology and Evolutionary Biology, University of California, Irvine, Irvine, CA 92697, USA; Department of Ecology and Evolutionary Biology, University of California, Irvine, Irvine, CA 92697, USA

**Keywords:** epistasis, gene expression, gene coexpression modules, rho termination, diminishing returns

## Abstract

Epistasis is caused by genetic interactions among mutations that affect fitness. To characterize properties and potential mechanisms of epistasis, we engineered eight double mutants that combined mutations from the *rho* and *rpoB* genes of *Escherichia coli*. The two genes encode essential functions for transcription, and the mutations in each gene were chosen because they were beneficial for adaptation to thermal stress (42.2 °C). The double mutants exhibited patterns of fitness epistasis that included diminishing returns epistasis at 42.2 °C, stronger diminishing returns between mutations with larger beneficial effects and both negative and positive (sign) epistasis across environments (20.0 °C and 37.0 °C). By assessing gene expression between single and double mutants, we detected hundreds of genes with gene expression epistasis. Previous work postulated that highly connected hub genes in coexpression networks have low epistasis, but we found the opposite: hub genes had high epistasis values in both coexpression and protein–protein interaction networks. We hypothesized that elevated epistasis in hub genes reflected that they were enriched for targets of Rho termination but that was not the case. Altogether, gene expression and coexpression analyses revealed that thermal adaptation occurred in modules, through modulation of ribonucleotide biosynthetic processes and ribosome assembly, the attenuation of expression in genes related to heat shock and stress responses, and with an overall trend toward restoring gene expression toward the unstressed state.

## Introduction

Epistasis is caused by genetic interactions (or G × G interactions) among mutations, and it can be either positive or negative. Positive epistasis refers to interactions between mutations that result in higher fitness than expected based on the combined effects of the individual mutations; negative epistasis produces fitness effects that are lower than expected. These interactions are important because they shape the outcome and trajectory of the evolutionary process. Epistasis can, for example, create complex fitness landscapes with alternative adaptive peaks. In fact, some epistasis (namely reciprocal sign epistasis) is necessary for the existence of more than one peak (Wright 1931; Szendro et al. 2013; Bank 2022). Epistasis can also create historical contingencies by constraining the possible order and identity of mutations (Weinreich et al. 2006; Bank 2022; Batarseh et al. 2023).

Given its importance in evolution, a large body of research has characterized the dynamics and properties of epistasis. One well-substantiated epistatic effect is diminishing returns, a form of negative epistasis in which advantageous mutations are less beneficial on fitter genetic backgrounds (Griffing 1950; Jerison and Desai 2015). The causes of diminishing returns epistasis are still not clear, but they likely result from a combination of factors, such as biases in the magnitude and direction of epistatic effect (Kryazhimskiy et al. 2014; Bank 2022). They may also reflect that two beneficial mutations can together overshoot a fitness optimum, leading to a negative fitness effect.

Unfortunately, fitness measurements alone do not typically provide insights into the molecular and genetic mechanisms that contribute to epistasis nor to the pathways or genes that are affected by epistatic interactions. The resulting lack of information on mechanisms remains a wide gap in our understanding of the causes and consequences of epistasis (Lehner 2011). To help fill this gap, researchers have turned to measuring epistatic interactions on phenotypes, such as protein folding, metabolic flux, and gene expression (reviewed in Domingo et al. 2019). These phenotypic measures often reflect features of fitness and can, in theory, uncover some of the mechanistic bases and molecular effects of epistatic interactions. One example illustrates the point: Boj et al. (2010) measured gene expression in double mutants of two transcription factor genes. They showed that the two transcription factors regulate similar sets of downstream genes and hypothesized that epistasis was caused by interactions between the transcription factors at common target sites.

In this work, we focus on characterizing epistasis between mutations identified in a large evolution experiment (Tenaillon et al. 2012). The experiment consisted of 115 populations that evolved from a single *Escherichia coli* founder strain (REL1206) under 42.2 °C thermal stress. After 2,000 generations of evolution, genome sequencing of one clone from each population revealed >1,300 total mutations. Many of these mutations were found independently across populations, providing strong evidence for beneficial effects. Moreover, some pairs of beneficial mutations were associated more (or less) often within clones than expected based on their observed frequencies. One overarching theme was negative associations between mutations in the RNA polymerase (RNAP) subunit beta (*rpoB*) gene and the transcriptional terminator (*rho*) gene, because mutations in these genes were found together within a single clone less often than expected. The results suggest that mutations in *rpoB* and *rho* define alternative evolutionary pathways, an idea supported by chemical phenotypes (Hug and Gaut 2015), trade-off dynamics across temperatures (Rodríguez-Verdugo et al. 2014), and adaptive genetic trajectories (Batarseh et al. 2023).

The observation of negative associations between *rho* and *rpoB* mutations is particularly interesting in the context of function. *rpoB* encodes the beta subunit of RNAP, a five-subunit protein that has the catalytic activity for RNA synthesis (Ebright 2000; Ishihama 2000). Evolution experiments have consistently identified adaptive genetic changes within the genes that encode the RNAP complex (Herring et al. 2006; Conrad et al. 2010; Sandberg et al. 2014; Deatherage et al. 2017), perhaps in part because RNAP mutations have the potential to modify the expression of every gene. That is, modifications to RNAP may be a potent, but not specific, instrument of adaptive change. Not surprisingly, studies of four of the putatively beneficial *rpoB* mutations from the Tenaillon et al (2012) experiment showed that they conferred large fitness benefits and altered the expression of thousands of downstream genes (Rodríguez-Verdugo et al. 2016).

Similar to RNAP complex genes, evolution experiments have also consistently identified adaptive genetic changes within the *rho* gene (Freddolino et al. 2012; Herron and Doebeli 2013; Dillon et al. 2016; Deatherage et al. 2017; Kinnersley et al. 2021; McGuire and Nano 2023), which encodes the Rho transcriptional terminator. Unlike RNAP, however, Rho does not act on all genes, because it terminates transcription for a subset of ∼20% to 50% of *E. coli* genes (Cardinale et al. 2008; Peters et al. 2009; Adams et al. 2021). Consequently, *rho* mutations are likely to have fewer downstream effects on gene expression than mutations in RNAP complex genes. Consistent with this idea, a study of four *rho* mutations from the Tenaillon experiment affected the expression of up to 1,000 genes (González-González et al. 2017), but the *rpoB* mutations altered the expression of roughly double that number (Rodríguez-Verdugo et al. 2016). The mutations in the two genes had overlapping effects, because 83% of the genes altered by *rho* mutations were also altered by *rpoB* mutations (González-González et al. 2017).

Although the Tenaillon experiment uncovered substantial evidence for negative associations between the two genes, the pattern and magnitude of epistatic effects have not been measured. In this study, we use site-directed mutagenesis to construct eight double mutants, all in the same genetic background (REL1206), with varying combinations of six putatively beneficial *rho* and *rpoB* mutations. With the double mutants in hand, we address four sets of questions. First, based on fitness effects, do double mutants exhibit diminishing returns epistasis? If so, does the magnitude of epistasis follow a general pattern? Second, since previous work has established that epistasis varies by environment (i.e. G × G × E interactions) (Wei and Zhang 2019; Kemble et al. 2020), does the magnitude and direction of epistasis in our system vary across temperatures? Third, does gene expression vary among single and double mutants, and do these differentially expressed genes (DEGs) provide insights into the mechanisms that lead to adaptation to thermal stress? Finally, we measure gene expression epistasis for all genes in the genome by comparing expression levels between double and single mutants. We then place gene expression epistasis in the context of coexpression networks and ask the question: Are highly connected genes differentially affected by epistasis? We pursue this last question because previous work in yeast has suggested that epistasis is less common for highly interconnected “hub” genes within molecular networks (Park and Lehner 2013; Alam et al. 2016). If the relationship between epistasis and hub genes is universal, it may have broad applicability for understanding the mechanistic bases of epistasis.

## Results

### Diminishing Returns at 42.2 °C

**Fig. 1.**
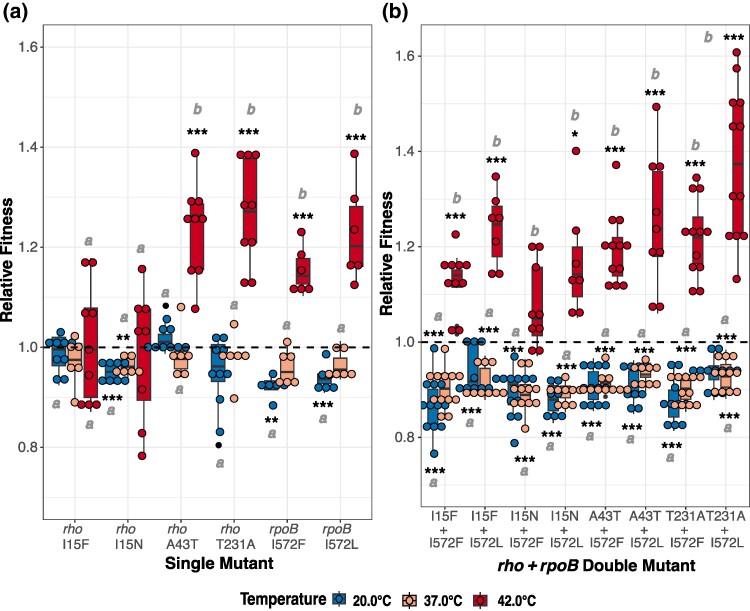
Relative fitness effects of *rho* and *rpoB* single mutations and *rho* + *rpoB* double mutants at different temperatures. a) Relative fitness values $\left({\bar{w}}_{r}\right)$ of *rho* and *rpoB* single mutants at 20.0 °C, 37.0 °C, and 42.2 °C. b) Relative fitness values $\left({\bar{w}}_{r}\right)$ of *rho* + *rpoB* double mutants at 20.0 °C, 37.0 °C, and 42.2 °C. For both a) and b), boxplots show the measurements of the relative fitness of each mutant in competition with the ancestral strain REL1207. Asterisks represent rejection of the null hypothesis $\left({\bar{w}}_{r}=0\right)$ represented by a dashed line, indicating that the mutants or double mutants were beneficial or deleterious relative to the ancestor at 20.0 °C, 37.0 °C, or 42.2 °C. ***P < 0.001, **P < 0.01, *P < 0.05, and •0.05 < *P* < 0.1. *P*-values were adjusted using the Bonferroni method (supplementary tables S1 and S2, Supplementary Material online). Words *a* and *b* denote if $\left({\bar{w}}_{r}\right)$ is significantly different among 20.0 °C, 37.0 °C, and 42.2 °C.

To examine the potential for epistasis between double mutants, we first gathered relative fitness (*w_r_*) values for six single mutants at 20.0 °C, 37.0 °C, and 42.2 °C. The six single mutants included four *rho* mutations (*rho*_I15N, *rho*_I15F, *rho*_A43T, and *rho*_T231A) and two *rpoB* mutations (*rpoB*_I572F and *rpoB*_I572L) that were engineered previously into the ancestral *E. coli* REL1206 background (Rodríguez-Verdugo et al. 2016; González-González et al. 2017). All $w_{r}$ values were measured against REL1207, an Ara^+^ version of the REL1206 (Ara^−^) ancestral background (see Materials and Methods). Generally, the assays showed that single mutations were advantageous (i.e. ${\bar{w}}_{r}$ > 1.0) at 42.2 °C but deleterious (i.e. ${\bar{w}}_{r}$ < 1.0) at 37.0 °C and 20.0 °C (Fig. 1a; supplementary table S1, Supplementary Material online). Two notable exceptions were the *rho*_*I15N* and *rho*_*I15F* mutations, which tended to be neutral (i.e. ${\bar{w}}_{r}$ = 1.0) at 42.2 °C, prompting the idea that they likely were advantageous only in combination with additional interacting mutations (González-González et al. 2017).

We also constructed eight double mutant combinations of *rho* and *rpoB* mutations to measure epistasis (Table 1). We assayed $w_{r}$ for double mutants with 243 fitness assays, representing both technical and biological replicates across double mutants at 20.0 °C, 37.0 °C, and 42.2 °C (see Materials and Methods). These assays revealed that each double mutant was advantageous at 42.2 °C, with an average fitness of ${\bar{w}}_{r}$ = 1.21 across all samples (Fig. 1b; supplementary table S2, Supplementary Material online). Variation in ${\bar{w}}_{r}$ was significant and fell into three overlapping groups (supplementary fig. S1, Supplementary Material online). At the lower temperatures (37.0 °C and 20.0 °C), each double mutant had significantly lower fitness than the ancestor (Fig. 1b; supplementary table S2, Supplementary Material online). Note that we also attempted to construct a ninth double mutant containing *rho*_I15N and *rpoB_*I966S, which were the most commonly observed mutations in the thermal stress experiment (Tenaillon et al. 2012). However, our several attempts failed.

**Table 1:** Epistatic deviation ($\varepsilon_{wr}$) of *rho* + *rpoB* double mutants calculated using relative fitness (${\underline{w}}_{r}$) estimates

Mutant	42.2 °C		37.0 °C		20.0 °C	
	$\varepsilon_{wr}$ ^a^ (Var)	*P* ^b^	$\varepsilon_{wr}$ ^a^ (Var)	*P* ^b^	$\varepsilon_{wr}$ ^a^ (Var)	*P* ^b^
***rhoA*43T + *rpoB*I572F**	−0.2325 (0.0182)	<0.000	−0.0319 (0.0033)	0.116	−0.0283 (0.0020)	0.0508
***rho*A43T + *rpo*BI572L**	−0.2680 (0.0418)	0.0043	−0.0171 (0.0031)	0.383	−0.0468 (0.0023)	0.0188
***rho*T231A + *rpoB*I572F**	−0.2430 (0.0218)	0.0001	−0.0430 (0.0034)	0.036	−0.0024 (0.0053)	0.9194
***rho*T231A + *rpoB*I572L**	−0.1802 (0.0505)	0.0180	−0.0155 (0.0034)	0.401	0.0377 (0.0045)	0.0772
***rho*I15F + *rpoB*I572F**	−0.037 (0.0215)	0.4431	−0.0304 (0.0045)	0.146	−0.0425 (0.0045)	0.0509
***rho*I15*F* + *rpoB*I572L**	−0.001 (0.0318)	0.9866	−0.0224 (0.0033)	0.281	0.0078 (0.0037)	0.7137
***rho*I15N *+ rpoB*I572F**	−0.0635 (0.02874)	0.2938	−0.0318 (0.0027)	0.073	0.0184 (0.0029)	0.2600
***rho*I15N + *rpoB*I572L**	−0.0446 (0.0430)	0.5898	−0.0360 (0.0014)	0.020	−0.0118 (0.0018)	0.4721

^a^Absolute epistatic deviation and its variance. $\varepsilon_{wr}$ > 0 and $\varepsilon_{wr}$ < 0 suggest positive and negative epistatic interactions between *rho* and *rpoB* mutations, respectively. ^b^Two-tailed *P* < 0.05 rejects the null hypothesis *ɛ* = 0.

**Fig. 2.**
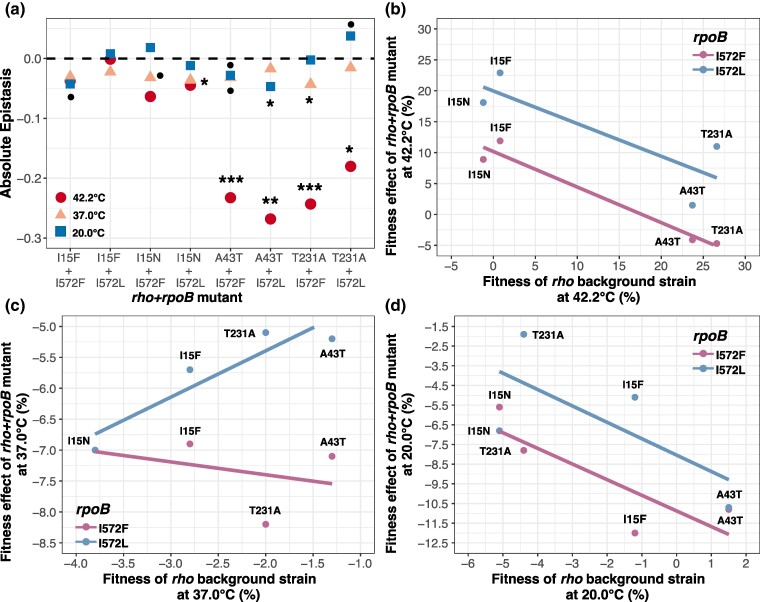
Patterns and magnitude of epistasis based on relative fitness $\left({\bar{w}}_{r}\right)$ at different temperatures. a) Epistatic interactions between *rho* and *rpoB* mutations at different temperatures. Epistasis values $\varepsilon_{wr}$ were estimated for each *rho* + *rpoB* double mutant using relative fitness measurements of the mutants in competition with the ancestor REL1207 at 42.2 °C, 37.0 °C, and 20.0 °C. Asterisks represent rejection of the null hypothesis $\varepsilon_{wr}=0$, represented by a dashed line. If $\varepsilon_{wr}>0$, epistatic interactions between *rho* and *rpoB* mutations are positive whereas a value of $\varepsilon_{wr}<0$ suggests negative epistasis. ***P < 0.001, **P < 0.01, *P < 0.05, and •0.05 < *P* < 0.1 (supplementary table S3, Supplementary Material online). b) An analysis exploring diminishing returns epistasis at 42.2 °C. The *y* axis plots the relative fitness effects of the *rpoB* mutations against the fitness effects of *rho* mutations on the *x* axis. The upper and lower lines refer to the effects of I572F and I572L *rpoB* mutations, respectively. The remaining panels are as b) except at 37.0 °C c) and 20.0 °C d).

We used ${\bar{w}}_{r}$ from single and double mutants to test for epistatic interactions based on a multiplicative null model (Khan et al. 2011). The model estimated the epistatic deviation with the metric $\varepsilon_{wr}$, which is expected to be 0 in the absence of epistasis (see Materials and Methods). There was negative epistasis between *rho* and *rpoB* single mutants (Table 1), particularly at 42.2 °C, where the average across all double mutants was ${\bar{\varepsilon }}_{wr}$ = −0.134 ± 0.090 (95% confidence interval [CI]) (*t*_7_ = −3.324, *P* = 0.010). However, double mutants that included *rho*_I15F and *rho*_I15N did not exhibit significant $\varepsilon_{wr}$ at 42.2 °C (Table 1; supplementary table S3, Supplementary Material online; Fig. 2a). Thus, the magnitude of $\varepsilon_{wr}$ varied among double mutants (Fig. 2a). We also compared epistasis across temperatures to assess environmental variation. On average, $\varepsilon_{wr}$ for double mutants was negative and significant at 37.0 °C (${\bar{\varepsilon }}_{wr}$ = −0.028 ± 0.008 [95% CI], *t*_7_ = −8.51, *P* = 6.13 × 10^−5^), but the effect was smaller and not significant at 20.0 °C (${\bar{\varepsilon }}_{wr}$ = −0.008 ± 0.025 [95% CI], *t*_7_ = −0.81, *P* = 0.446) (supplementary tables S3 to S5, Supplementary Material online). The magnitude and distribution of $\varepsilon_{wr}$ varied significantly across the three temperatures (*F*_2,21_ = 8.603, *P* = 0.002). Most of this variation was due to shifts in the magnitude, but not the direction, of $\varepsilon_{wr}$ across environments. However, sign epistasis occurred in at least one case, because $\varepsilon_{wr}$ was positive for *rho*_T231A + *rpoB*_I572L at 20.0 °C ($\varepsilon_{wr}$ = 0.0377; *P* = 0.0772) despite being negative at 42.2 °C ($\varepsilon_{wr}$ = −0.1802, *P* = 0.018) (Fig. 2a).

In addition to a multiplicative model, we evaluated diminishing returns following a previous approach that compares the relative fitness of single mutants to double mutants (Kryazhimskiy et al. 2014). Since we inserted the *rpoB* single point mutations (I572F or I572L) into *rho* single mutants, we measured the fitness effect of each single *rpoB* mutation on different *rho* backgrounds. Under diminishing returns, we expected the more fit *rho* single mutants to lead to smaller fitness gains. As expected, we detected a significantly negative linear relationship (negative slope) between the fitness effects of the combined set of I572F and I572L *rpoB* mutations and the fitness of single *rho* mutants at 42.2 °C (slope = −1.024, *P* = 0.031; Fig. 2b). We also detected a combined negative slope at 20.0 °C (slope = −0.549, *P* = 0.069; Fig. 2d); the significance of the slope was borderline but the pattern at this temperature was similar to the expectation under diminishing returns. In contrast, at 37.0 °C, we detected a positive correlation across all data (slope = 0.228, *P* = 0.552), but the slopes diverged substantially between *I572L rpoB* and *rpoB I572F* mutations, neither of which was significant by itself (*P* > 0.05) (Fig. 2c). We conclude that diminishing returns epistasis holds in some but not all of our three environments, pointing to G × G × E effects.

Finally, we measured growth rate, a key phenotype, of the *rho + rpoB* double mutants at 37.0 °C and 42.2 °C (supplementary fig. S2, Supplementary Material online). At 42.2 °C, all *rho + rpoB* double mutants reached lower final yields than the ancestor at 37.0 °C. Nonetheless, all but one (*rho*_I15N *+ rpoB*_I572L) had significantly higher yield and higher maximum growth rate at 42.2 °C compared to the ancestor (supplementary fig. S2 and tables S6 and S7, Supplementary Material online). Generally, these analyses indicate that the high temperature was still stressful, given that the double mutants did not grow as rapidly as the ancestor at 37 °C, but they also verified the beneficial effects of double mutants.

### Gene Expression Dynamics of Double Mutants at 42.2 °C

**Fig. 3.**
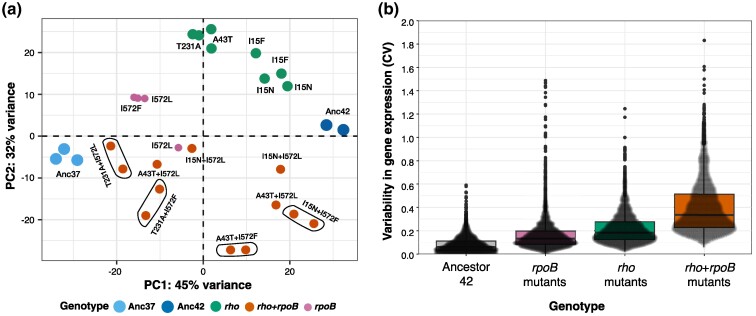
Gene expression of *rho* and *rpoB* single mutations and *rho* + *rpoB* double mutants at 42.2 °C. a) PCA plot showing the first two principal components of the RNAseq data of four different *rho* and two different *rpoB* single mutants as well as from six *rho + rpoB* double mutants at 42.2 °C. Gene expression profiles of the ancestor at 37.0 °C and 42.2 °C are also included. Circled dots represent replicates from the same labeled genotype. b) Shift in gene expression variability present in *rho + rpoB* double mutants compared to *rho* and *rpoB* single mutants at 42.2 °C. Variability in gene expression was measured as the coefficient of variation (CV in normalized read count) for each one of 4,114 genes across four *rho* single mutants, two *rpoB* mutants, and six *rho + rpoB* double mutants and the Ancestor at 42.2 °C. Boxplots show the interquartile range and median of each data distribution.

To explore the effects of epistatic interactions between *rho* and *rpoB* mutations, we constructed an RNA-sequencing (RNAseq) data set. The data set included replicates of six double mutants at 42.2 °C, six single mutants at 42.2 °C, and control data from REL1206 at two temperatures (37.0 °C and 42.2 °C). Similar to single mutants (Rodríguez-Verdugo et al. 2016; González-González et al. 2017), double mutants at 42.2 °C tended to restore gene expression toward the unstressed (37.0 °C) state, as reflected in principal component analysis (PCA) space. The double and single mutants were intermediate between the two ancestors on PC1, which explained 45% of the expression variance (Fig. 3a). The double mutants further adjusted expression patterns along PC2, which explained 32% of variance. Gene expression varied more markedly across the set of double mutants than within single mutants, as measured by the coefficient of variation of expression for individual genes (Fig. 3b).

We next identified DEGs compared to REL1206 grown at 42.2 °C. We detected 2,700 DEGs (*q* < 0.001) across all six double mutants. The number varied across double mutants, ranging from 127 (for *rho_*A43T *+ rpoB_*I572L) to 2,110 (for *rho_*T231A *+ rpoB_*I572L), likely representing both biological and experimental differences among samples (supplementary fig. S3, Supplementary Material online). One way to characterize expression shifts is to count the number of DEGs in four alternative categories of directional change, reflecting genes that had restored, unrestored, novel, or reinforced expression (see Materials and Methods) (Carroll and Marx 2013). Most genes in double mutants were expressed at levels between the stressed and unstressed ancestor, indicating that the mutation(s) acted to restore expression partially or fully to the unstressed state (supplementary fig. S4, Supplementary Material online), as indicated by their location on PC1 and PC2 (Fig. 3a). We note two additional trends among DEGs. First, DEGs (*q* < 0.001) were significantly overrepresented for upregulation, as opposed to downregulation (supplementary fig. S3, Supplementary Material online) in all six double mutants but *rho_*T231A *+ rpoB_*I572F. For example, *rho*_A43T + *rpoB*_I572F had 849 upregulated versus 530 downregulated genes, with similar results for *rho*_A43T + *rpoB*_I572L (101 vs. 26), *rho*_I15N + *rpoB*_I572F (608 vs. 527), *rho*_I15N + *rpoB*_I572L (154 vs. 56), and *rho*_T231A + *rpoB*_I572L (1,238 vs. 880). Second, 99 DEGs (*q* < 0.001) (3.7%) were shared across all six double mutants, making them especially strong candidates for adaptive shifts in gene expression. Gene ontology (GO) enrichment analysis of these 99 genes revealed enrichment for bacterial-type flagellum-dependent cell motility (GO: 0071973, *P* = 1.33 × 10^−5^), bacterial-type flagellum assembly (GO: 004478, *P* = 4.47 × 10^−2^), ribonucleotide biosynthetic process (GO: 0009260, *P* = 2.46 × 10^−6^), and ribosome assembly (GO: 0042255, *P* = 3.13 × 10^−11^). There were fewer downregulated genes, but they were significantly enriched for genes involved in galactose catabolic processes (GO: 0019388, *P* = 5.21 × 10^−3^) (supplementary table S8, Supplementary Material online).

Similar trends were evident with stronger filtering criteria. For example, when we filtered for DEGs (*q* < 0.001) and fold-change differences (|>2.0|), there were 660 DEGs identified across the six double mutants. These again tended to be upregulated more often than downregulated relative to the REL1206 ancestor (*rho*_A43T + *rpoB*_I572F, 178 upregulated vs. 73 downregulated genes; *rho*_A43T + *rpoB*_I572L, 99 vs. 23; *rho*_I15N + *rpoB*_I572F, 46 vs. 33; *rho*_I15N + *rpoB*_I572L 65 vs. 26; *rho*_T231A + *rpoB*_I572F, 203 vs. 154; and *rho*_T231A + *rpoB*_I572L, 274 vs. 215), but with a smaller subset of 29 DEGs shared among all double mutants. These 29 DEGs were enriched for genes associated with “de novo” pyrimidine nucleobase biosynthetic process (GO: 0006207, *P* = 4.79 × 10^−3^), bacterial-type flagellum-dependent cell motility (GO: 0071973, *P* = 4.26 × 10^−13^), and bacterial-type flagellum assembly (GO: 004478, *P* = 3.05 × 10^−3^).

Finally, we focused on the expression of sets of genes with a priori functional interest. We first focused on genes that contribute to transcriptional machinery. The *rho* gene and some components of the RNAP (*rpoA*, *rpoB*, *rpoC*, and *rpoZ* genes) were often upregulated relative to the ancestor in some double mutants, but the effect was rarely significant for individual genes (supplementary table S9, Supplementary Material online). The second gene set was the heat shock genes, which can play a role in acclimatization and adaptation to heat stress at 42.2 °C (Riehle et al. 2003). On average, ∼20% of previously identified heat shock-induced genes in *E. coli* (Riehle et al. 2003; Nonaka et al. 2006; Gunasekera et al. 2008) were differentially expressed in each double mutant, but the percentage was notably higher, at ∼40% (on average), for single mutants (supplementary fig. S5, Supplementary Material online). Thus, the global expression of heat shock genes was attenuated in double mutants compared to single mutants (supplementary fig. S5 and Data Set S1, Supplementary Material online). Finally, we investigated a set of 65 genes that were previously identified to be involved in the *E. coli* stress response network (Weber et al. 2005). A few double mutants (*rho_*A43T + *rpoB*_1572F, *rho_*T231A + *rpoB*_1572L, and *rho_*T231A + *rpoB*_1572L) exhibited significant changes in the expression of ∼35% of these genes, and these were typically downregulated (supplementary fig. S5 and Data Set S1, Supplementary Material online). These genes also had decreased expression in *rpoB* single mutants and two of the four *rho* single mutants (A43T and T231A) (supplementary fig. S5 and Data Set S1, Supplementary Material online).

### The Extent and Pattern of Gene Expression Epistasis

We used RNAseq data to measure epistasis in individual genes (Angeles-Albores et al. 2018) by comparing expression levels between two single mutants and their corresponding double mutants. Following precedence (Phillips et al 2000; Baier et al. 2023; Behr et al. 2024), we calculated epistasis using an additive model (Fisher 1919; Alam et al. 2016). The additive model is similar to the multiplicative model used to evaluate fitness epistasis, given that we measured gene expression on a logarithmic scale. In this model, $\varepsilon_{wr}$ can again be either positive or negative.

**Fig. 4.**
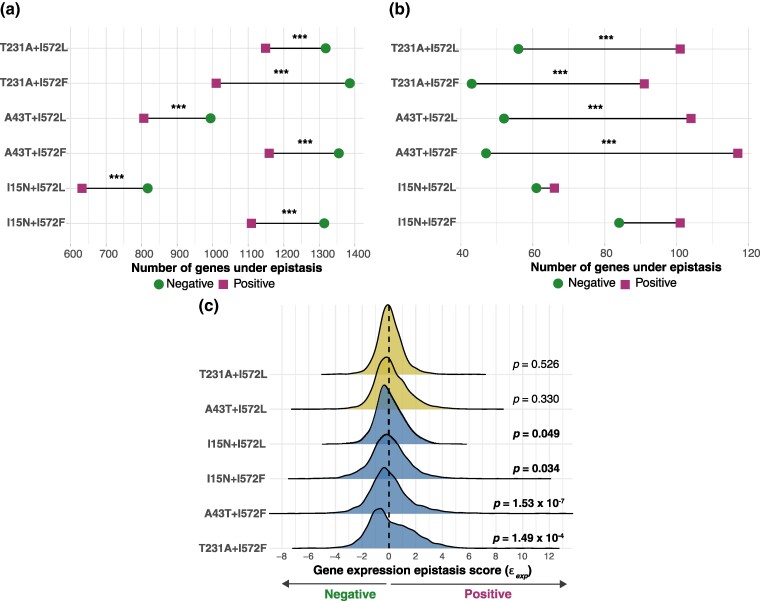
Epistatic interactions using genome-wide transcriptome profiling $\left(\varepsilon_{\exp}\right)$. Number of genes exhibiting positive $\left(\varepsilon_{\exp}>0\right)$ and negative $\left(\varepsilon_{\exp}<0\right)$ epistatic interactions per *rho + rpoB* mutant. a) Lollipop plot showing the number of genes with positive and negative epistatic scores (that were DEGs [*q* < 0.001] in at least one mutant [*rho*, *rpoB*, or *rho + rpoB* mutant]). b) Lollipop plot showing the number of genes with highly significant epistatic effects—*q* < 0.001, log_2_-fold change > 2 or <−2—in at least one mutant (*rho*, *rpoB*, or *rho + rpoB* mutant) and an epistatic standard score (*Z*-score) of >2σ or <−2σ. For both a) and b), asterisks represent statistical difference (binomial test) between the number of genes under positive and negative epistasis. ***P < 0.001 and **P < 0.01. Note that the colors flip between a) and b), showing that there are more or less positively epistatic genes depending on the filtering criteria used. c) The distribution of $\left(\varepsilon_{\exp}\right)$ scores for all genes in the genome of the six double mutants studied. *P*-values represent statistical difference (binomial test) between the number of genes under positive and negative epistasis.

We summarized global patterns of $\varepsilon_{wr}$ in two ways. First, we examined the distribution across individual genes, based on genes that were differentially expressed (*q* < 0.001) in at least one mutant (*rho* or *rpoB* or *rho* + *rpoB*) compared to the ancestor at 42.2 °C. Significantly more of this gene set had negative $\left(\varepsilon_{\exp}<0\right)$ than positive epistasis $\left(\varepsilon_{\exp}>0\right)$ across all six double mutants (binomial test *P*: *rho*_*I15N + rpoB*_*I572*F = 3.37 × 10^−5^, *rho*_*I15N + rpoB*_*I572L* = 1.30 ×10^−6^, *rho*_*A43T + rpoB*_*I572*F = 9.99 × 10^−5^, *rho*_*A43T +rpoB*_*I572L* = 1.03 × 10^−5^, *rho*_*T231A + rpoB*_*I572F* =1.63 × 10^−14^, and *rho*_*T231A + rpoB*_*I572L* = 7.15 × 10^−4^) (Fig. 4a). The trend was not robust, however. For example, when we considered genes that were more stringently filtered (i.e. *q* < 0.001 and fold-change > |2| in at least one mutant plus a standard $\varepsilon_{\exp}$  *Z*-score > 2σ or <−2σ; see Materials and Methods), there were significantly fewer negatively $\left(\varepsilon_{\exp}<0\right)$ than positive $\left(\varepsilon_{\exp}>0\right)$ epistatic genes (binomial test *P*: *rho*_*I15N + rpoB*_*I572*F = 0.240, *rho*_*I15N + rpoB*_*I572L* = 0.723, *rho*_*A43T + rpoB*_*I572*F = 4.50×10^−8^, *rho*_*A43T + rpoB*_*I572L* = 3.81 × 10^−5^, *rho*_*T231A +rpoB*_*I572F* = 4.11 × 10^−5^, and *rho*_*T231A + rpoB*_*I572L* = 4.11 × 10^−4^) (Fig. 4b). From these stringently filtered genes, we identified 54 that were shared across all double mutants, of which 32 and 22 exhibited positive and negative ɛ_exp_, respectively. Among this gene set, positively epistatic genes were enriched for functions in maltose transmembrane transport (*lamB* and *mal*; GO: 1904981, *P*-adjusted =6.24 × 10^−3^) and the transmembrane transport of an array of organic substances (GO: 0071702, *P*-adjusted = 8.28 ×10^−3^) (supplementary fig. S6, Supplementary Material online). In contrast, the ɛ_exp_ < 0 genes were enriched for translation (*rpl* and *rps*; GO: 0006412, *P*-adjusted = 8.99 ×10^−8^) and sulfur compound biosynthetic processes (*bio* and *met*; GO: 0044272, *P*-adjusted = 7.68 × 10^−8^) (supplementary fig. S6, Supplementary Material online).

Second, we calculated the mean epistasis score $\left({\bar{\varepsilon }}_{\exp}\right)$ across all genes in the genome. ${\bar{\varepsilon }}_{\exp}$ values were >0.0 in all *rho* + *rpoB* mutants, with most statistically significant (two-tailed *t*-test of ${\bar{\varepsilon }}_{\exp}$ = 0; supplementary table S10, Supplementary Material online). This observation resulted from positively skewed and heavy-tailed (leptokurtic) $\varepsilon_{\exp}$ distributions for all six *rho + rpoB* double mutants (Fig. 4c). The most extreme example was *rho*_*A43T + rpoB*_*I572F*, which had the most positive skew ($\gamma_{1}$ = 1.184) and a particularly heavy tail ($\gamma_{2}$ = 9.078) (supplementary table S10, Supplementary Material online). In contrast to ${\bar{\varepsilon }}_{\exp}$, median $\varepsilon_{\exp}$ was negative for four of six double mutants (range −0.05 to −0.15) and only slightly positive for two (0.02 and 0.01).

### Coexpression Networks and the Distribution of Epistasis

Previous work has suggested that highly connected genes are less likely to be affected by epistasis because they typically have more stable gene expression (Park and Lehner 2013; Alam et al. 2016). To investigate this idea in our system, we constructed coexpression modules from 29 RNAseq libraries (see Materials and Methods) (supplementary fig. S7, Supplementary Material online). Using a soft thresholding power of 16, we identified seven gene modules that included 92% (3,844 out of 4,174) of the analyzed transcripts (supplementary fig. S7, Supplementary Material online). The modules, which by convention we refer to by different colors, grouped different numbers of genes. For example, the turquoise module contained the highest proportion of genes (39.9%; 1,534 genes out of 3,844) while the black module had the lowest (4.16%; 160 genes out of 3,844) (Table 2). As expected, the modules varied substantially in gene expression (supplementary fig. S8, Supplementary Material online) and also differed with respect to functional enrichments (supplementary fig. S8, Supplementary Material online). Notable observations include that the turquoise module was enriched for functions related to DNA recombination, nucleotide metabolic processes, RNA processing, transcription, translation, and iron, antibiotic, and bacteriocin transport; the black module contained flagellum organization and flagellum-dependent motility genes as well as genes that respond to external stimuli; the yellow module was enriched for response to heat and sulfur metabolism; and the green module was enriched for genes that regulate transcription and transmembrane transport (supplementary fig. S8, Supplementary Material online).

**Table 2:** Significant gene expression epistasis $\left(\varepsilon_{\exp}\right)$ and hub genes per module

Module^a^	Total number of genes^b^	Number of module genes under epistasis (%)^c^	*k* within (mean ± SE module genes under epistasis)^d^	Number of hub genes^e^	*k* within (mean ± SD hub genes)^f^	Hub genes under epistasis (%)^g^
Turquoise	1,534	184 (11.99)	32.59 ± 1.69	152	61.67 ± 0.76	46 (30.26)
Blue	919	49 (5.33)	20.73 ± 2.40	92	45.50 ± 0.67	12 (13.04)
Brown	373	12 (3.21)	14.78 ± 3.37	37	39.69 ± 0.81	1 (2.70)
Green	260	80 (30.76)	10.69 ± 0.67	26	18.41 ± 0.61	20 (76.92)
Black	160	18 (11.25)	16.38 ± 1.58	16	20.58 ± 0.27	11 (68.75)
Red	248	2 (0.81)	1.47 ± 1.24	25	9.01 ± 0.29	0 (0)
Yellow	350	61 (17.43)	7.41 ± 0.58	35	11.57 ± 0.25	27 (77.14)

^a^Modules were detected by hierarchical clustering using the R WGCNA package (see Materials and Methods). ^b^Number of genes clustered per module. ^c^Number (and percentage) of genes significantly epistatic per module in at least one double mutant. We used the following criteria to define significance: genes differentially expressed in any mutant (*q* < 0.001) and that present an epistatic standard score (*Z*-score) of >2σ or <−2σ. ^d^Average and standard deviation of the intramodular connectivity (or degree) of the genes that are under significant epistasis per module. Intramodular connectivity for each individual gene was calculated using the *intramodularConnectivity* function in the R WGCNA package. ^e^Hub genes in each module were identified as those with high connectivity (i.e. intramodular connectivity within the top 10% of all gene members of the module), a modular membership > 0.9 and high significance (*P* < 0.01). ^f^Average and standard deviation of the intramodular connectivity (or degree) of the hub genes that are under significant epistasis per module. ^g^Number (percentage) of hub genes that are classified as significantly epistatic in at least one double mutant under the following criteria: genes differentially expressed in any mutant (*q* < 0.001) and that present an epistatic standard score (*Z*-score) of >2σ or <−2σ.

**Fig. 5.**
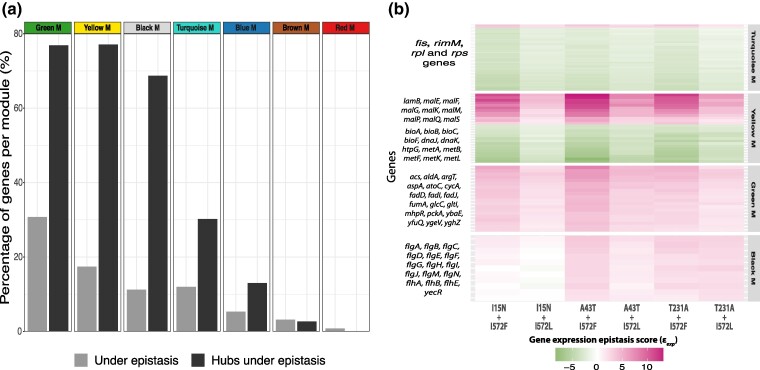
Gene expression epistasis within modules and hub genes. a) Shows the percentage of genes significantly epistatic in at least one double mutant (light gray bars) and the percentage of hub genes that are classified as significantly epistatic in at least one double mutant (dark gray bars) per module. b) Heatmap showing the gene expression epistasis scores of the significantly epistatic hub genes per module (selected modules), along with the names of some representative genes on the *y* axis. Epistasis significance was defined as being differentially expressed (*q* < 0.001) in at least one *rho*, *rpoB*, or *rho + rpoB* mutant and present an epistatic standard score (*Z*-score) of >2σ or <−2σ.

We also investigated the distribution of significant $\varepsilon_{\exp}$ values (DEG in any mutant *q* < 0.001 plus a $\varepsilon_{\exp}$  *Z*-score of >2σ or <−2σ in at least one double mutant) among the genes of each module (supplementary fig. S9, Supplementary Material online; Table 2). The Green module had the highest proportion (30.8%) of significantly epistatic genes, followed by yellow (17.4%), black (11.2%), and turquoise (11.99%) modules (Table 2; Fig. 5a). We then tested the idea that hub genes have lower epistasis by defining hub genes as having module membership assignment > 0.9, strong statistical significance for module membership (*P* < 0.01), and intramodular connectivity among the top 10% of all members in each module. We identified a total of 383 hub genes across all modules (Table 2, supplementary Data Set S2, Supplementary Material online). The direction of epistasis varied across modules (Fig. 5b;  supplementary fig. S10, Supplementary Material online). Most hubs within the blue, brown, green, and black modules had $\varepsilon_{\exp}>0$ in all double mutants, while the Yellow and Red modules had a mix of hub genes with both $\varepsilon_{\exp}>0$ and $\varepsilon_{\exp}<0$. Notably, nearly all hub genes from the turquoise module, which have roles related to core functions like transcription and translation, were negatively epistatic in all double mutants (Fig. 5b). The yellow and green modules had the highest proportion of significant epistatic hub genes (yellow module: 77.1%, 27 genes; green module: 76.9%, 20 genes), followed by the black (68.75%, 11 genes) and turquoise modules (30.3%, 46 genes). In contrast, the brown and red modules had the lowest proportion (brown module: 2.7%, 1 gene; red module: 0%) of hub genes with significant ɛ_exp_ (Table 2).

**Fig. 6.**
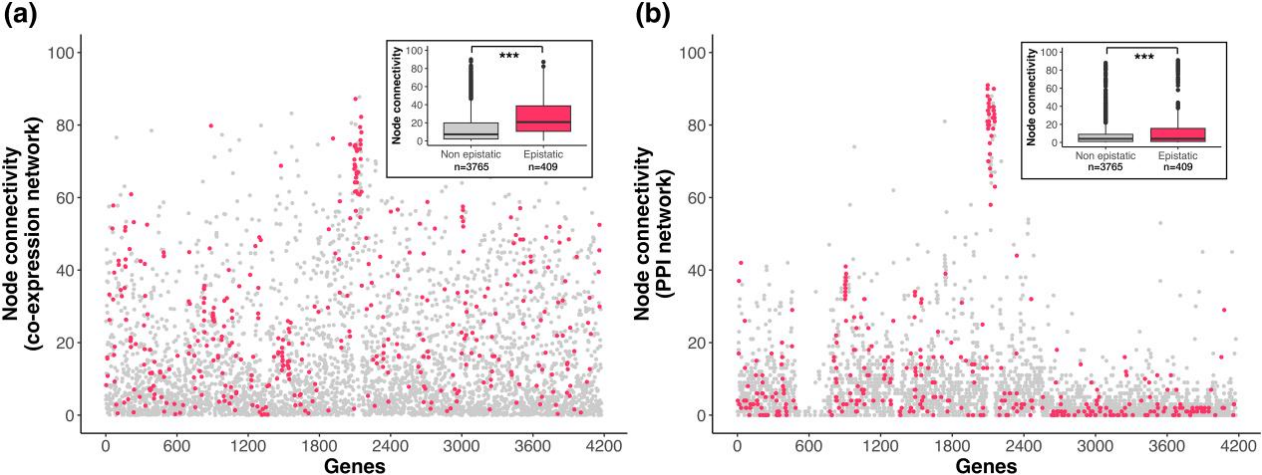
Connectivity levels of epistatically responding transcripts. Connectivity levels (node degree) distribution of the *E. coli* genes that exhibit significant epistatic interactions (darker pink dots) and all other genes (light gray dots) in gene coexpression network a) and in a Protein–Protein interaction network b). Genes were sorted alphabetically on the *x* axis. Significant epistatic genes were considered as those being differentially expressed (*q* < 0.001) in at least one *rho*, *rpoB*, or *rho + rpoB* mutant and that presented an epistatic standard score (*Z*-score) of >2σ or <−2σ. Asterisks in the boxplots represent statistical difference (Wilcoxon test) between the connectivity levels of epistatic and nonepistatic transcripts. ***P < 0.001.

We then compared $\varepsilon_{\exp}$ to node connectivity, based on both our coexpression networks and protein–protein interaction networks (see Materials and Methods). Unlike previous work (Alam et al. 2016), we found that genes with significantly epistatic transcript levels were more connected in coexpression networks (epistatic genes: mean = 26.25, median = 20.77, nonepistatic genes: mean = 13.60, median = 7.26; *P* < 2.2 × 10^−16^) (Fig. 6a) and also had more protein–protein interactions (epistatic genes: mean = 14.29, median = 4, no epistatic genes: mean = 6.94, median = 4; *P* = 8.7 × 10^−6^) than non-epistatic genes (Fig. 6b).

We hypothesized that $\varepsilon_{\exp}$ and node connectivity could be related to Rho termination. We hypothesized that Rho-terminated genes have lower $\varepsilon_{\exp}$, due to the potential interactions and functional overlaps between Rho and RNAP, since both play a role in transcription termination (Das et al. 1978; Jin et al. 1992). To investigate this idea, we filtered a list of ∼1,000 putatively Rho-terminated genes (Adams et al. 2021) to find 743 genes present in REL1206. Only 70 of these genes (1%) had $\varepsilon_{\exp}$ significantly different from 0. Across all 743 genes, more had $\varepsilon_{\exp}$ < 0 compared to $\varepsilon_{\exp}$ > 0, but the difference was significant for only one double mutant (*rho_*I15N + *rpoB_*I572L; binomial, *P* = 0.0016; supplementary fig. S11, Supplementary Material online). We also evaluated if the ɛ_exp_ scores of Rho-terminated genes differed from the rest of the genome. Although ${\bar{\varepsilon }}_{\exp}$ was lower for the set of rho-terminated genes compared to the nonrho-terminated gene set across five out of six double mutants (*rho_*A43T + *rpo*B_I572F had similar ${\bar{\varepsilon }}_{\exp}$ values for both set of genes; ${\bar{\varepsilon }}_{\exp}$ = 0.75), in only one double mutant, the difference was significant (*rho_*I15N + *rpo*B_I572L: $\varepsilon_{\exp}$ rho-terminated genes = −0.06 vs. $\varepsilon_{\exp}$ non-rho terminated-genes = 0.12; Wilcoxon test; *P* = 0.0003). We further hypothesized that Rho-terminated genes had lower variance in ɛ_exp_ for Rho- and non-Rho-terminated genes, reflecting tighter expression control. While the variance was consistently lower for Rho-terminated genes, it was borderline significant for only *rho_*T231A + *rpoB_*I572F (*F*-test; *P* = 0.038). Finally, we compared the proportion of Rho-terminated genes in hubs; 6.5% of Rho-terminated genes were classified as hub genes, which did not reflect a significant enrichment (Fishers Exact Test; *P* = 0.21). Thus, we found no obvious patterns that connect Rho termination to $\varepsilon_{\exp}$ or to network connectivity.

## Discussion

We have measured epistasis between mutations in the *E. coli rho* and *rpoB* genes. The mutations were first identified in an evolution experiment as potentially beneficial for high-temperature adaptation (Tenaillon et al. 2012). None of the mutations cause a loss of function, because *rho* and *rpoB* are essential genes that regulate transcription. Because the mutations alter the expression of numerous downstream genes, they represent a particularly interesting system to measure epistasis at the level of gene expression. To our knowledge, genome-wide gene expression epistasis has not been measured previously for *E. coli*.

### Fitness Epistasis Varies across Mutations and Environments

One prominent conclusion from empirical studies is that beneficial alleles often interact negatively, leading to diminishing returns epistasis (Chou et al. 2011; Khan et al. 2011; Tenaillon et al. 2012; Kryazhimskiy et al. 2014). In this study, we expected diminishing returns between *rho* and *rpoB* mutations at 42.2 °C based on their patterns of repulsion in the thermal stress experiment (Tenaillon et al. 2012). Negative epistasis was most obvious for the two *rho* mutations (*rho* A43T and T231A) that were detectably beneficial at 42.2 °C as single mutants (Fig. 1a) (González-González et al. 2017). Double mutants with these *rho* mutations still had a fitness advantage at 42.2 °C relative to the ancestor, but at levels ∼10% to 20% reduced relative to the multiplicative prediction (Fig. 1b). The two other *rho* mutations in this study (*rho_*I15N and *rho*_I15F) conferred no significant advantage on their own (Fig. 1a) (Tenaillon et al. 2012; González-González et al. 2017). Of these, one (*rho_*I15N) demonstrated significant negative epistasis with *rpoB* mutations (Fig. 1b), but negative interactions were less dramatic for a second mutation in the same codon (*rho*_I15F) (Fig. 2a). Taken together, our results demonstrate that the presence and magnitude of negative epistasis are common in this system and that it follows a general pattern (Wang et al. 2016), because mutations with larger beneficial effects had stronger (i.e. more negative) epistatic interactions (Fig. 2b).

An interesting feature of *rho* and RNAP is that they modulate and affect gene expression. Given their importance, one might expect that mutations in both genes could overtune expression, leading to highly deleterious fitness effects. This may occur for some combinations of mutations, because we were unable to engineer viable clones containing the two most frequently observed alleles (*rho*_I15N and *rpoB_*I966S) from the Tenaillon et al (2012) experiment. Although one must be careful not to overinterpret based on potential lab failures, our results are consistent with the fact that these two mutations did not co-occur in the thermal stress experiment despite being within the most commonly mutated codons (Tenaillon et al. 2012). We thus tentatively conclude that their co-occurrence leads to synthetic lethality in the REL1206 background.

Transcription function was not severely disrupted for the remaining double mutants, because they remained beneficial at 42.2 °C (Figs. 1a and 2a). This causes one to wonder why some *rho + rpoB* double mutants are, or are not, evolutionary dead ends. We suspect that part of the answer to this question lies in redundancy. We have previously shown that (i) the effect of single mutations in both *rpoB* and *rho* act to restore global gene expression from the stressed state back toward the unstressed state, (ii) mutations in *rho* and *rpoB* affect overlapping sets of genes, but (iii) mutations in *rpoB* tend to affect the expression of more genes (Rodríguez-Verdugo et al. 2016; González-González et al. 2017). Under this model, sufficient restoration of expression to enhance fitness may be mostly achieved by single mutants, such that additional mutations do not contribute as dramatically (and with less benefit) to restoring gene expression. Consistent with this model, we find that double mutants had similar numbers of genes with restored expression compared to single *rpoB* mutants (supplementary fig. S4, Supplementary Material online). In fact, 53% of the genes with restored expression in all six double mutants also showed patterns of restored expression in both *rho* and *rpoB* single mutants. Another 38% of the restored genes (321/838) were shared only with *rpoB* mutants. We recognize that redundancy does not explain the apparent lethality of the *rho*_I15N + *rpoB_*I966S combination, suggesting idiosyncratic exceptions to these general patterns. Thus, our work contributes to the growing consensus that diminishing returns are generally predictable but with idiosyncratic exceptions that likely reflect properties of both individual mutations and the fitness landscape (Wei and Zhang 2019; Lyons et al. 2020; Card et al. 2021; Bakerlee et al. 2022; Couce et al. 2024).

Epistasis represents genetic (G × G) interactions, but it can also vary with environment (G × G × E). We previously assessed fitness across three temperatures for the single mutants and found they were either neutral or significantly deleterious at 20.0 °C or 37.0 °C (Fig. 1a; supplementary fig. S2, Supplementary Material online) (Rodríguez-Verdugo et al. 2016; González-González et al. 2017). Epistasis was typically slightly (but significantly) negative for most *rho + rpoB* pairs at these two lower temperatures (Fig. 2a), but there were exceptions, such as two double mutants with estimated positive epistasis at 20.0 °C (Fig. 2a). What is most apparent, however, is that the magnitude and pattern of epistasis varied across environments, with especially large negative interactions at 42.2 °C. G × G × E variation was evident because of reduced slopes between the high- and low-temperature environments (Fig. 2b and d) and a divergent pattern at 37.0 °C (Fig. 2c). Although these relationships appear to be idiosyncratic, they fit themes found in other empirical work, which is that the direction of epistatic interactions tend to be similar across environments, but not always, and that the magnitude of G × G effects is often higher in more extreme environments (Flynn et al. 2013; Ono et al. 2017; Ghenu et al. 2023).

### Gene Expression Shifts in Double Mutants

By comparing gene expression to the REL1206 ancestor, we identified 99 DEGs shared across all six double mutants. Most (97%) of these 99 genes overlapped with DEGs from single mutants, with only three (*iraP*, *yobD*, and *cpxP*) unique to double mutants. One of these, *yobD*, is annotated as a membrane-bound protein with a hypothesized role in mannose operon (Pan et al. 2021). The second, *iraP* (previously known as *yaiB*), is upregulated in the ancestral background at 42 °C (compared to 37 °C), so the double mutants partially restore expression of this gene when the single mutants do not. At least one of the evolved lines from Tenaillon et al. (2012) had additional mutations that also reversed expression of *iraP* (Rodríguez-Verdugo et al. 2016), suggesting that restoring *iraP* expression is a repeatable component of thermal adaptation. *IraP* modulates the stability of the sigma stress factor RpoS (Bougdour et al. 2008), which in turn regulates the expression of a suite of genes involved in stress responses (Loewen and Hengge-Aronis 1994). The third gene, *cpxP*, is a periplasmic protein that acts in an envelope stress response system as a signaling protein and as a proteolytic adaptor that degrades some misfolded proteins (Thede et al. 2011).

Our measurement of $\varepsilon_{\exp}$ has documented that gene expression epistasis is rampant. Each double mutant had from 600 to 1,400 genes with significant epistasis (i.e. $\varepsilon_{\exp}$ ≠ 0.0) that were also differentially expressed (*q* < 0.001) between at least one mutant and the ancestor at 42.2 °C (Fig. 4a). Among these, 54 were epistatic in all six double mutants, of which 13 (24%) overlapped with the set of common 99 DEGs. Of those 13, ten were *rpl* and *rps* genes. The remaining three were *tnaA* that encodes a tryptophanase that produces the signaling molecule indole (Lee and Lee 2010), *uidC* (also known as *gusC*) that encodes a membrane-associated protein involved in glucuronide transport (Liang et al. 2005), and *ydjN* that encodes an L-cystine transporter purported to have a role in defense against oxidative stress (Ohtsu et al. 2015). The remaining 41 of 54 genes included eight genes involved in maltose transport (Davidson and Nikaido 1991) and six genes involved in methionine biosynthesis like *metA*, which affects thermal tolerance (Mordukhova and Pan 2013).

Overall, gene expression analyses have revealed major themes related to thermal adaptation. The first is that adaptive mutations have the overarching effect toward restoring gene expression from a stressed to a less stressed state. This occurs for the single mutants and for the double mutants, and it is a common phenomenon during adaptation (Ho and Zhang 2018). Interestingly, restoration is also linked to gene expression epistasis, because restored genes are overwhelmingly enriched for negative ɛ_exp_ values across all six double mutants (binomial, *P* < 0.01; supplementary fig. S12, Supplementary Material online). A second theme relates to gene function, including upregulation of flagellar genes (Rodríguez-Verdugo et al. 2016), the alteration of expression for genes that affect ribonucleotide biosynthetic processes and ribosome assembly, and a general attenuation of expression for genes related to heat shock and stress responses.

### Gene Expression Epistasis Preferentially Affects Hub Genes in *rho* + *rpoB* Double Mutants

Highly connected hub genes tend to be essential (Jeong et al. 2001) with larger effects on phenotypes (Batada et al. 2006), lower evolutionary rates (Fraser et al. 2002), and expression patterns with low stochastic and environmental variance (Park and Lehner 2013). It has also been argued that the conserved expression of hub genes is related to negative epistasis (Park and Lehner 2013). The idea is that negative epistasis amplifies the deleterious effects of expression variance between two interacting partners; hence, selection will act to reduce gene expression variability in genes with many interacting partners. The prediction of low expression variance for hub genes was supported by analyses of yeast data (Park and Lehner 2013). Subsequent work measured gene expression epistasis in yeast and concluded it was more prominent “…in the least connected and conserved genes, while highly conserved genes and most connected genes appear to be buffered…” against gene expression epistasis (Alam et al. 2016).

Our results differ from these previous results. In fact, we observe the opposite: hub genes tend to exhibit *more* epistasis in our system based on the analysis of both coexpression and protein–protein interaction networks (Fig. 6). What can resolve the difference between our study and previous studies? One potential explanation is that patterns of epistasis vary between systems; yeast and *E. coli* differ in numerous important genetic attributes that could influence patterns of epistasis, including ploidy number, overall genomic architecture, and methods of controlling gene expression. Another general explanation is that the expectation of low $\varepsilon_{\exp}$ in hub genes may be incorrect. Boross and Papp (2017) investigated this expectation using both empirical analyses of large-scale expression data and biochemical modeling. They found that hub genes did have lower expression noise and also that negative epistasis was a negligible predictor of expression variability. Their biochemical modeling further showed that gene expression epistasis is unlikely to amplify the fitness cost of variation in gene expression. Thus, while hub genes do appear to be under selection for low expression variance (Park and Lehner 2013; Alam et al. 2016; Boross and Papp 2017), epistasis may not play a consequential role in shaping that variance. We also hypothesized that our observations might be unique to our system, particularly if hub genes were enriched for Rho-terminated genes. Under this model, functional interactions between Rho and RNAP in these genes would generate negative epistasis. Unfortunately, this explanation yielded no insights, because we detected no notable $\varepsilon_{\exp}$ trends related to Rho-terminated genes. Of course, direct interactions between Rho and RNAP may generate gene expression epistasis, but if they do, the signal does not stand out against the genome-wide background.

### Functional Insights into Thermal Adaptation

The coexpression modules reinforce many of our observations based on gene expression analyses, but it remains challenging to bridge the gap between fitness and $\varepsilon_{\exp}$. For example, the turquoise module is enriched for genes that function in transcription, translation, and recombination, along with iron transport. Moreover, its hub genes are universally under negative epistasis. Based on the function of the mutated genes, gene expression shifts in the module, and consistent signals of negative $\varepsilon_{\exp}$, it seems likely that many of the genes in this module could contribute to negative fitness epistasis in the double mutants. But which subset of genes contribute directly to fitness, or do all, or do none? Similarly, the green module has the highest proportion of epistatic genes, at 77%, and these genes also encode transcriptional functions (supplementary fig. S8, Supplementary Material online). In contrast, the black module contains many of the upregulated flagellar genes, which are largely under positive epistasis. There is now ample evidence from evolution experiments that modified expression of flagellar genes can and do affect fitness (Rodríguez-Verdugo et al. 2016; Rychel et al. 2024), probably by shunting resources between mobility and growth. There is also evidence, somewhat counterintuitively, that the upregulation of basal flagellar genes can affect envelope stability or protein export (Rychel et al. 2024), meaning that the upregulation of a subset of these genes could be beneficial at high temperatures. Altogether, it is difficult to conclude that there is a strong relationship between negative $\varepsilon_{\exp}$ (as in transcription-related genes), positive $\varepsilon_{\exp}$ (as in many flagellar genes), and either fitness or fitness epistasis. However, one thing is clear: epistasis between *rho* and *rpoB* mutations affect diverse sets of genes, suggesting that genes with many functions—from transcription to stress response to flagellar components—likely contribute to thermal adaptation.

## Materials and Methods

### Construction of *rho* + *rpoB* Double Mutants

We constructed eight *rho + rpoB* double mutants by introducing the *rpoB* single mutations I572F and I572L into the four different *rho* single mutants (I15F, I15N, A43T, and T231A) that had been engineered previously (González-González et al. 2017) . Briefly, we first introduced the pJk611 plasmid into each *rho* single mutant by electroporating 2 µl of the plasmid (at 33 ng/µl) into 50 µl of electrocompetent cells using an Eppendorf Electroporator 2510 set at 1.8 kV. Electrocompetent cells of each *rho* mutant were obtained by washing each culture five times with ice-cold water. Immediately after electroporation, 1 ml of LB medium was added to electroporated cells and they were incubated for 2 h at 30.0 °C under constant shaking at 120 rpm. Then, 100 µl of electroporated cells were plated onto LB + ampicillin 100 µg/ml agar plates in order to select for those cells that incorporated the plasmid pJk611.

Cultures of each *rho* mutant containing pjK611 were grown overnight at room temperature in 25 ml of LB supplemented with 100 µg/ml of ampicillin and 1 mM L-arabinose (Sigma) until they reached a final density of 0.06 OD_600_. Competent cells of each *rho* mutant were obtained by washing the cultures five times with ice-cold water. To construct the double *rho* + *rpoB* mutants, we introduced the single point mutations I1572F or I572N in the *rpoB* gene into the *rho* mutants carrying the pkJ611 plasmid. We did this by electroporating 10 µM (2.3 µl) of each 70-bp *rpoB* oligo into 50 µl of electrocompetent cells. After electroporation, we added 1 ml of fresh LB and incubated cells at 37.0 °C for 3 h at constant shaking at 120 rpm, and then, we left them incubating overnight at room temperature. The next day we plated 100 µl of the electroporated cells 50:50 diluted onto LB agar plates containing rifampicin 50 µg/ml and left them growing overnight at 37.0 °C. We selected single colonies and performed colony PCR followed by Sanger sequencing of ∼420 bp of the *rpoB* gene to confirm the correct base replacement. We used primers and PCR conditions described in Rodríguez-Verdugo et al. (2014). For each double mutant, we isolated four different colonies with the desired mutation, which we consider independent biological replicates. Altogether, we constructed eight double mutants, representing all possible combinations between the two *rpoB* and the four *rho* single mutants.

### Relative Fitness Assays and Analyses

We performed competition assays between each double mutant and the ancestral Ara + REL1207 strain to obtain estimates of the mean relative fitness ${\bar{w}}_{r}$ as previously described (Lenski et al. 1991; González-González et al. 2017). The assays were performed at three different temperatures, 20.0 °C, 37.0 °C, and 42.2 °C, for at least three biological replicates for each double mutant and for three technical replicates per biological replicate. Following precedence (Tenaillon et al. 2012; Rodríguez-Verdugo et al. 2014), the two competitors were revived separately by growing them overnight in LB broth at 37.0 °C under constant shaking at 120 rpm. Then, the overnight cultures were diluted 100-fold in saline solution and 100 µl of this dilution was transferred into 9.9 ml of fresh DM25 and incubated at 37.0 °C under constant shaking at 120 rpm in order to acclimate from frozen conditions. After 24 h, the cultures were 100-fold diluted by transferring 100 µl of the acclimated cultures into 9.9 ml of fresh DM25 and then incubated at the temperature of interest (20.0 °C, 37.0 °C, and 42.2 °C) at constant shaking (120 rpm). The following day, the ancestor and double mutant were mixed at a 1:1 ratio. This mixture was plated onto tetrazolium–arabinose (TA) agar plates and incubated at the temperature of interest (20.0 °C, 37.0 °C, or 42.2 °C) to obtain the initial cell densities of the ancestor REL 1207 (white colonies) and the double mutants (red colonies). Additionally, 100 µl of this 1:1 mixture was added to 9.9 ml of fresh DM25 and incubated at the temperature of interest (20.0 °C, 37.0 °C, or 42.2 °C) at constant shaking (120 rpm) for 24 h to let the ancestor and double mutant to compete. To quantify the cell densities after competition, this culture was plated onto TA agar plates and incubated at the temperature of interest (20.0 °C, 37.0 °C, or 42.2 °C). The initial and final number of ancestor and double mutant colonies was analyzed as in Lenski et al. (1991) and Tenaillon et al. (2012). We also obtained the mean relative fitness $\left({\bar{w}}_{r}\right)$ at 20.0 °C and 37.0 °C of all four *rho* single mutants (three biological replicates, two experimental replicates per biological replicate) by competing each *rho* mutant against the ancestor REL1207 as described above.

We tested for differences among biological replicates within single and double mutants by running an ANOVA using the *avo* package in R (Team RC 2013). We found no differences among biological replicates, and therefore, we ran the model fitness ∼ mutant for further analyses. To test for significant differences between the ${\bar{w}}_{r}$ values against the null hypothesis of ${\bar{w}}_{r}$ = 1.0, we performed a two-tailed *t*-test. To detect if ${\bar{w}}_{r}$ differed across all single mutants and all *rho* + *rpoB* mutants, we ran ANOVA and post hoc analyses using the *avo* and *agricolae* (de Mendiburu 2019) packages in R. We ran independent sample, two-tailed *t*-tests to evaluate if the fitness effects of each single and double mutant depend on the environment (20.0 °C, 37.0 °C, or 42.2 °C).

### Absolute Fitness and Growth Rate Measurement

To obtain the growth parameters of double mutants, we acclimatized the strains by growing their frozen stocks in LB broth at 37.0 °C overnight followed by another day of growth in DM25 media at 37.0 °C (Lenski and Bennett 1993). We grew the acclimated double mutants in DM25 media for 1 d at the assay temperature (either 37.0 °C or 42.2 °C) and measured their cell densities every hour during the lag phase and every ∼15 min during the exponential phase. Cell densities were obtained using the Beckman Coulter Counter model Multisizer 3 equipped with a 30-µm diameter aperture in volumetric mode. We took electronic counts by diluting 50 µl of each culture into 9.9 ml of Isoton II diluent. We also took electronic counts of 50 µl of sterile DM25 and subtracted this background noise to the density counts. We estimated the growth parameters for each double mutant based on three independent replicates. We estimated the absolute fitness $\left({\bar{w}}_{a}\right)$ of each double mutant by obtaining the maximum growth rate, μ_max_. We fit a linear regression to the natural logarithm of the cell density over time during the linear component of the exponential phase using the *lm* function in R. The final yield per double mutant was estimated from the cell counts at the end of the exponential phase. We assessed statistical differences between the ancestor and double mutants or between each double mutant grown at 37.0 °C and 42.2 °C using two sample *t*-tests. Growth rate parameters of the ancestor and *rho* and *rpoB* single mutants at 37.0 °C and 42.2 °C were taken from (Rodríguez-Verdugo et al. 2016; González-González et al. 2017). Although the measurements of single versus double mutants were taken a few years apart, we used the same Coulter Counter instrument to collect the data and also repeated analysis of ancestors to compare growth rates to pertinent mutants measured in the same timeframe.

### Measuring Epistasis with Relative Fitness

To assess how the interactions among *rho* and *rpoB* mutations affect the relative fitness $\left({\bar{w}}_{r}\right)$ of the *rho* + *rpoB* double mutants, we calculated the epistatic deviation assuming a multiplicative model as follows:


(1) $$\varepsilon_{rxy}={\bar{w}}_{rxy}-{\bar{w}}_{rx}{\bar{w}}_{ry},$$

where ${\bar{w}}_{rxy}$, ${\bar{w}}_{rx}$, and ${\bar{w}}_{ry}$ correspond to the mean ${\bar{w}}_{r}$ of the double mutant and each single *rho* and *rpoB* mutant, respectively (da Silva et al. 2010; Khan et al. 2011). This equation measures the difference between the observed and the expected ${\bar{w}}_{r}$ of a double mutant. The variance associated with the epistatic deviation was obtained by using the equation to calculate the variance of the product of independent random variables (Goodman 1962) as follows:


(2) $$\mathrm{Var}({\bar{w}}_{rx}{\bar{w}}_{ry})=[\left(\mathrm{Var}({\bar{w}}_{rx})+{\bar{w}}_{rx}^{2})\times (\mathrm{Var}({\bar{w}}_{ry})+{\bar{w}}_{ry}^{2}\right)]-{\bar{w}}_{rx}^{2}{\bar{w}}_{ry}^{2},$$

where $\mathrm{Var}({\bar{w}}_{rx})$ and $\mathrm{Var}({\bar{w}}_{ry})$ corresponds to the variance of a single *rho* and *rpoB* mutation respectively estimated from at least six replicate fitness assays (supplementary table S1, Supplementary Material online). Since the uncertainty of the epistatic deviation depends on both the uncertainty of the observed double mutant and the uncertainty of its expected fitness, the variance of epistasis was then estimated as the sum of both variances as follows:


(3) $$\mathrm{Var}(\varepsilon_{rxy})=\mathrm{Var}({\bar{w}}_{rxy})+\;\mathrm{Var}({\bar{w}}_{rx}{\bar{w}}_{ry}),$$

where $\mathrm{Var}({\bar{w}}_{rxy})$ refers to the variance of the observed double mutant's ${\bar{w}}_{r}$ estimated from at least seven fitness assays (supplementary table S2, Supplementary Material online) for the exact number of replicates per double mutant.

We used *t*-tests to test for significant epistatic deviations. We obtained the *t*-statistic by dividing the mean absolute epistasis by its standard error and the degrees of freedom based on the number of fitness assays replications of each *rho* + *rpoB* double mutant (Khan et al. 2011). In the absence of epistasis, the observed and expected ${\bar{w}}_{r}$ of a double *rho* + *rpoB* mutant equals 0 $\left(\varepsilon_{rxy}=0\right)$. The second term of Equation (3) assumes that the variances of the single mutants are independent, which could lead to an underestimate of the variance component if the model does not hold. This may affect tests of significance, but it does not affect estimates of the magnitude of $\varepsilon_{rxy}$.

### RNAseq and Analysis

We obtained the transcriptome of each double mutant to study the gene expression changes elicited by mutations. We harvested cells for RNAseq by reviving cells as mentioned above and then growing cultures of each double mutant at 42.2 °C until reaching the mid-exponential growth phase based on cell counts. We harvested two biological replicates per double mutant by vacuum filtrating 80 to 120 ml of bacterial culture through a 0.2-μm cellulose membrane (Life Science, Germany). We then washed the cells with Qiagen RNA-protect Bacteria Reagent, pelleted them and stored them at −80 °C. Prior to RNA extractions, pellets were thawed by incubating the cells with lysozyme (1 mg/ml) for 5 min at room temperature. Total RNA was isolated using the RNeasy Mini Kit (Qiagen), and DNA was removed by an on-column DNAse treatment performed at room temperature for 30 min. High-quality RNA extractions (RNA Integrity Number value above 9 assessed by running an Agilent RNA-Nano chip on a bionalyzer) were rRNA depleted by using the Ribo-Zero rRNA Removal kit for Gram-negative bacteria (Epicentre Biotechnologies, Medion, WI, USA). cDNA libraries were constructed using the TruSeq RNA v2 kit from Illumina (San Diego, CA, USA) and sequenced on an Illumina HiSeq 2000 system (100-bp single-end sequencing) at the UC Irvine Genomics High Throughput Facility. RNAseq data for rho and rpoB single mutations and the ancestor REL1206 were taken from (Rodríguez-Verdugo et al. 2016; González-González et al. 2017), which were generated using identical protocols to the ones described here. We generated RNAseq libraries from double mutants in duplicate, for a total data set of 29 RNAseq libraries that represented controls (ancestral), single mutants, and double mutants. The data are available at the NCBI's Sequence Read Archive (ancestor and *rpoB* data: BioProject Number PRJNA291128; *rho* data: BioProject Number PRJNA339971; *rho + rpoB* double mutants data: PRJNA945500).

RNAseq reads from both single and double mutants were filtered to a quality cut-off of 20 using a custom Perl script. The filtered reads were mapped to the *E. coli* B REL606 reference genome using BWA (Li and Durbin 2010) version 0.6.2 with default parameters. Counts of the uniquely mapping reads to each of the 4,204 *E. coli* B REL606 coding regions were obtained using the htseq-count script (-m intersection-nonempty) from HTSeq (Anders et al. 2015). PCA was performed using the *plotPCA* function from DESeq2 version 1.20.0 (Love et al. 2014) with default parameters (ntop = 500). Differential gene expression analysis was also performed using DESeq2 version 1.20.0 (Love et al. 2014). GO enrichment analysis with Bonferroni correction for multiple testing was performed online through the Gene Ontology Consortium webpage (http://geneontology.org; last accessed February 2022).

### Evolutionary Change

Changes in differential gene expression were classified into one of four directions of evolutionary change as previously described (Carroll and Marx 2013; Rodríguez-Verdugo et al. 2016; González-González et al. 2017). These directions represent the change of gene expression of a double mutant relative to the ancestor's gene expression at the stressed (42.2 °C) and unstressed (37.0 °C) temperatures. Briefly, a gene was classified as restored if it was differentially expressed (*q* < 0.001) between the ancestral treatments (Anc42 vs. Anc37) and between the mutant and the ancestor both grown at the stressed temperature (Mut42 vs. Anc42) and in an opposite direction to the one from the ancestral gene expression at 42.2 °C. A gene was reinforced if it was differentially expressed in the ancestral condition (Anc42 vs. Anc37) and in the mutant (Mut42 vs. Anc42) in the same direction to that of the ancestral gene expression at 42.2 °C. A gene had novel expression if its ancestral expression was not significantly different when grown at 42.2 °C and 37.0 °C (Anc42 vs. Anc37) but it was significantly different in a mutant compared to the ancestor at both temperatures (Mut42 vs. Anc42 & Mut42 vs. Anc37). Genes with unrestored gene expression were those for which gene expression change was significantly different in their ancestral state at 42.2 °C and 37.0 °C (Anc42 vs. Anc37) but nonsignificant in the mutant when contrasted to the ancestor at 42.2 °C (Mut42 vs. Anc42).

We obtained the coefficient of variation (CV = σ/μ) for each gene and each of 13 genotypes. To calculate the CV, we used a normalized transcript abundance matrix that included all biological replicates per genotype. The matrix was normalized using the size factors obtained after running the *estimateSizeFactors* function from DESeq2 version 1.20.0 (Anders and Huber 2010).

### Estimating Epistasis Using Transcription-Wide Expression Levels

We calculated an epistatic score $\left(\varepsilon_{\exp}\right)$ value for each gene following Fisher's additive (Alam et al. 2016) model as follows:


$$\varepsilon_{\exp}=GE_{xy}-(GE_{x}+\;GE_{y}),$$

where *GE_xy_*, *GE_x_*, and *GE_y_* refer to the gene expression change (log_2_-fold change) of a particular gene in the *rho + rpoB* double mutant, the *rho* and the *rpoB* single mutants respectively compared to the ancestor at 42.2 °C. When $\varepsilon_{\exp}>0$ and $\varepsilon_{\exp}<0$, and then positive and negative epistatic interactions are indicated, respectively, with respect to log_2_-fold expression change. To identify genes with epistatic behavior, we required both gene expression differentiation (i.e. *q* < 0.001 and log_2_-fold change > 2 or <−2) and significant deviation from the null hypothesis of no epistasis ($i.e.,\;\;\varepsilon_{\exp}=0$), as indicated by a *Z*-score of >2σ or <−2σ. Skewness and kurtosis (Pearson’s measure) of the epistatic scores distributions were estimated using the R package *moments*. We applied an additive model to gene expression data both to follow precedence (Phillips et al 2000; Baier et al. 2023; Behr et al. 2024) and because we used logarithmic expression counts.

### Coexpression Networks and Module Detection

We constructed a coexpression network and identified expression modules using the R package WGCNA (Langfelder and Horvath 2008). The normalized expression profiles of 29 RNAseq libraries were used to inferred a signed coexpression network, which were obtained by applying a variance stabilizing transformation to the count data (*varianceStabilizingTransformation* function) in DESeq2 version 1.20.0 (Anders and Huber 2010). We inferred a signed coexpression network with an optimal soft threshold = 16. Gene modules within the network were assigned using the topological overlap matrix. We also identified those modules that were significantly associated with relative fitness values at 20.0 °C, 37.0 °C, and 42.2 °C, and we extracted them for further analysis. Module networks were visualized using Cytoscape v3.8.2 software (Shannon et al. 2003). GO enrichment analyses were performed online through the Gene Ontology Consortium webpage (http://geneontology.org) to find out whether the genes composing each module are enriched in a particular biological function. We also identify key hub genes in each module as those with high connectivity (i.e. intramodular connectivity within the top 10% of all gene members of the module), a modular membership > 0.9 and high significance (*P* < 0.01).

We retrieved the protein–protein interaction network of all genes of the *E. coli* K12 substr. MG155 (4,127 nodes and 15,368 edges) genome from the STRING v.11.5 database (Szklarczyk et al. 2019) through the Cytoscape stringApp (https://apps.cytoscape.org/apps/stringapp). We obtained the number of interactions per each gene (node connectivity) by using the highest confidence score (0.9) as a cutoff for showing interaction links. We used EcoCyc *E. coli* Database (www.ecocyc.org) (Keseler et al. 2021) to map the *E. coli* K12 substr. MG155 gene names to the *E. coli* B REL606 genome.

## Supplementary Material

msae187_Supplementary_Data

## Data Availability

All of the RNAseq data used in this project are publicly available at the NCBI's Sequence Read Archive (ancestor and *rpoB* data: BioProject Number PRJNA291128; *rho* data: BioProject Number PRJNA339971; *rho + rpoB* double mutants data: PRJNA945500).
