# Supplementary material for: Patterns of Fitness and Gene Expression Epistasis Generated by Beneficial Mutations in the rho and rpoB Genes of Escherichia coli during High-Temperature Adaptation
Source: Mol Biol Evol. 2024 Sep 5;41(9):msae187. doi: 10.1093/molbev/msae187 (PMC11414761; doi:10.1093/molbev/msae187)
Supplement: msae187_Supplementary_Data [file msae187_supplementary_data.zip › MBE_SuppFigures_082624.pdf]

SUPPLEMENTAL FIGURES

Patterns of fitness and gene expression epistasis generated by beneficial mutations in the *rho* and *rpoB* genes of *Escherichia coli* during high-temperature adaptation

Andrea González-González, Tiffany N. Batarseh, Alejandra Rodríguez-Verdugo and Brandon S. Gaut

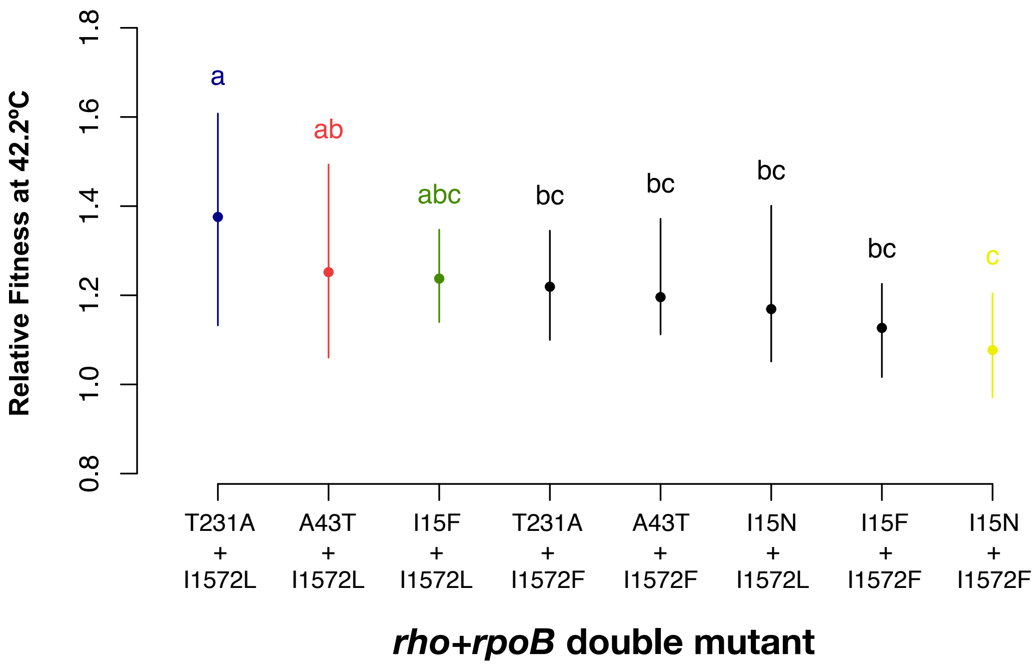

Figure S1: Relative fitness of *rho+rpoB* double mutants at 42.2°C. The plot shows the average, minimum and maximum relative fitness values per double mutant. The letters represent the result of Tukey-tests based on the *r* library *agricolae*. *rho+rpoB* double mutants with the same letter are not significantly different in fitness.

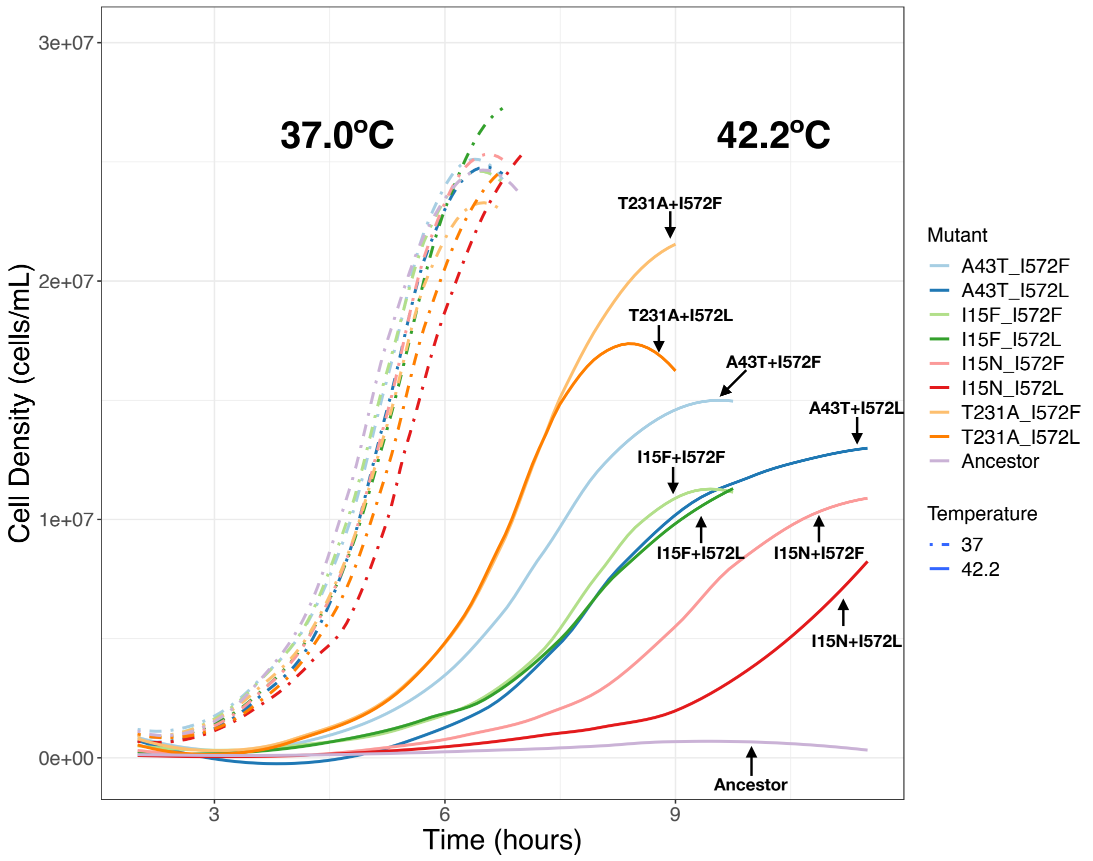

Figure S2: Growth curves for the *rho* and *rpoB* double mutants at 37.0°C (dashed lines) and 42.2°C (solid lines). Each double mutant is represented by a local polynomial regression fitting of three replicates.

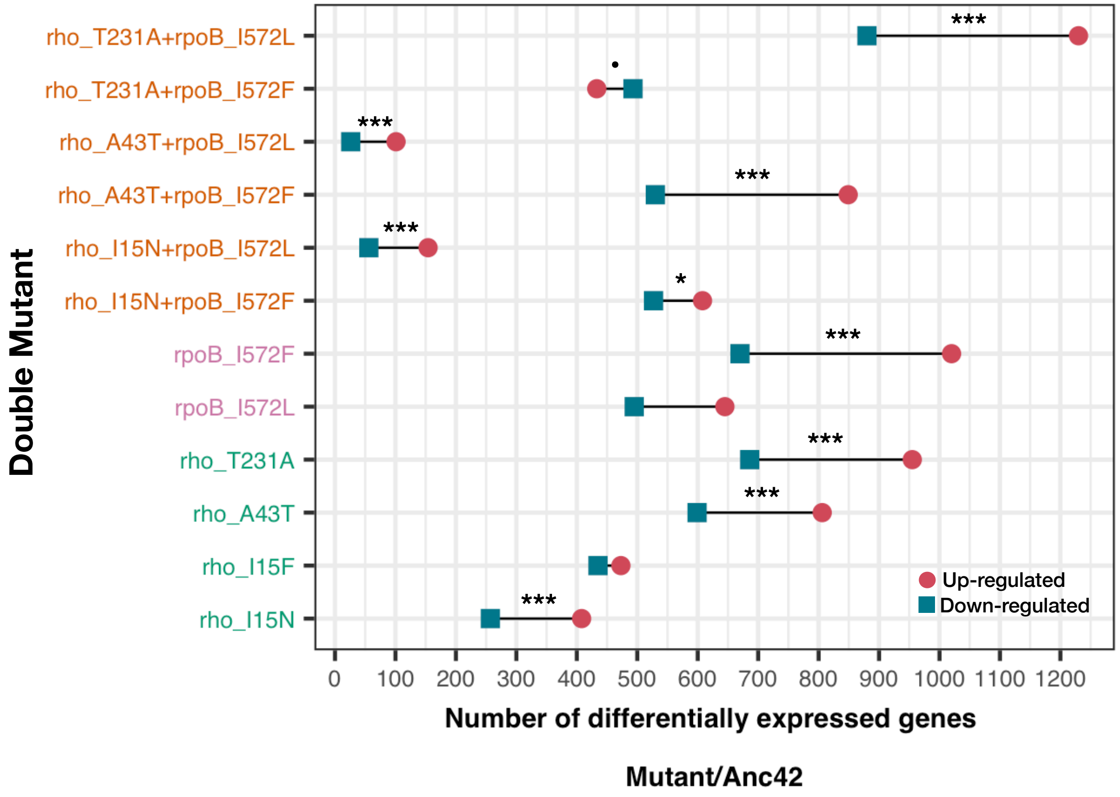

Figure S3: Gene expression changes of rho+rpoB double mutants at 42.2°C. Lollipop plot showing the number of differentially expressed genes ($q < 0.001$, down-regulated: blue squares, up-regulated: red-dots). Asterisks represent statistical difference (binomial test) between the number of up and down regulated genes. *** $P < 0.001$, ** $P < 0.01$ and, * $P < 0.05$. RNAseq data of *rho* and *rpoB* single mutants was obtained from (Rodríguez-Verdugo et al., 2014; González-González et al., 2017). RNAseq data of *rho+rpoB* mutants is from this study.

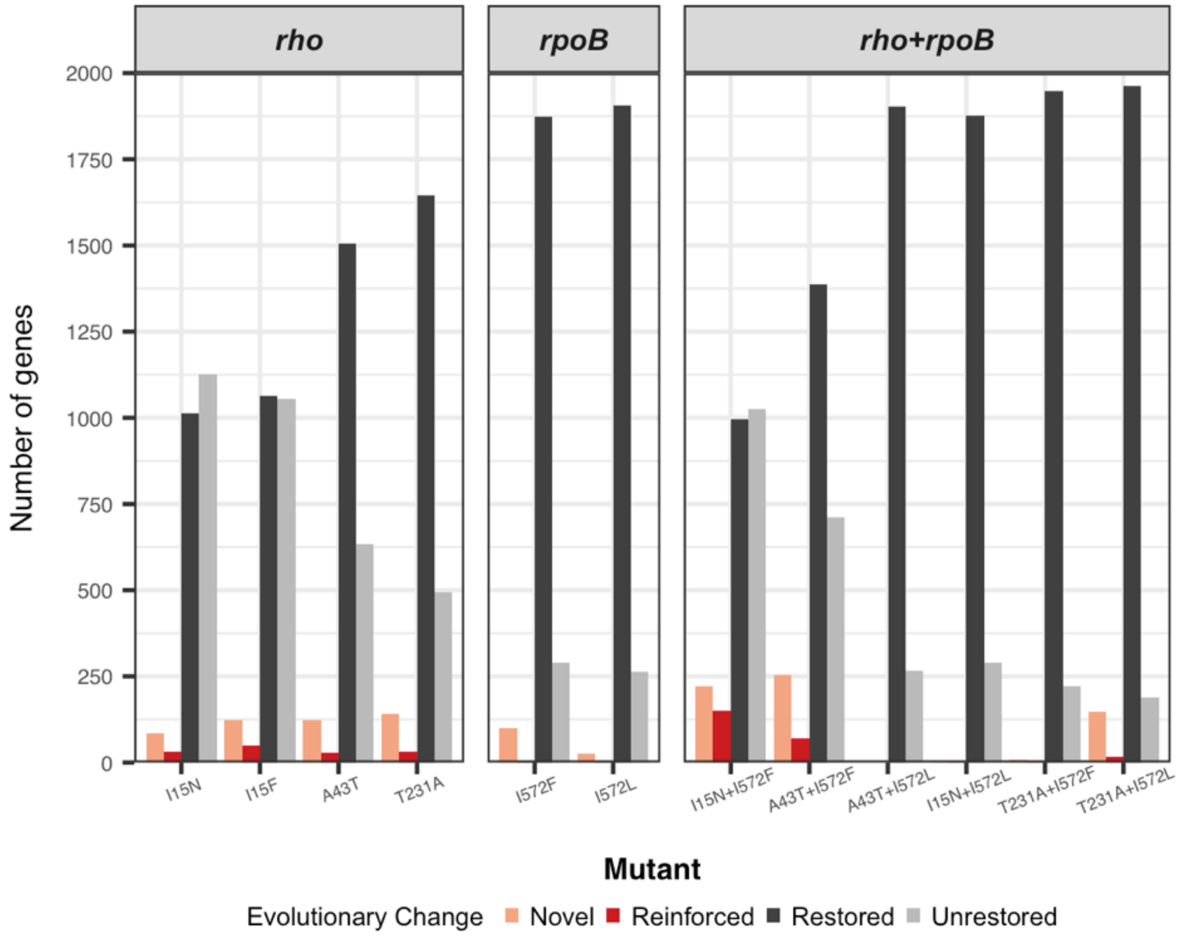

Figure S4: Barplots depicting the number of genes with restored (dark gray), unrestored (light gray) reinforced (red), and novel (light orange) expression within *rho+rpoB* double mutants compared to the *rho* and *rpoB* single mutants.

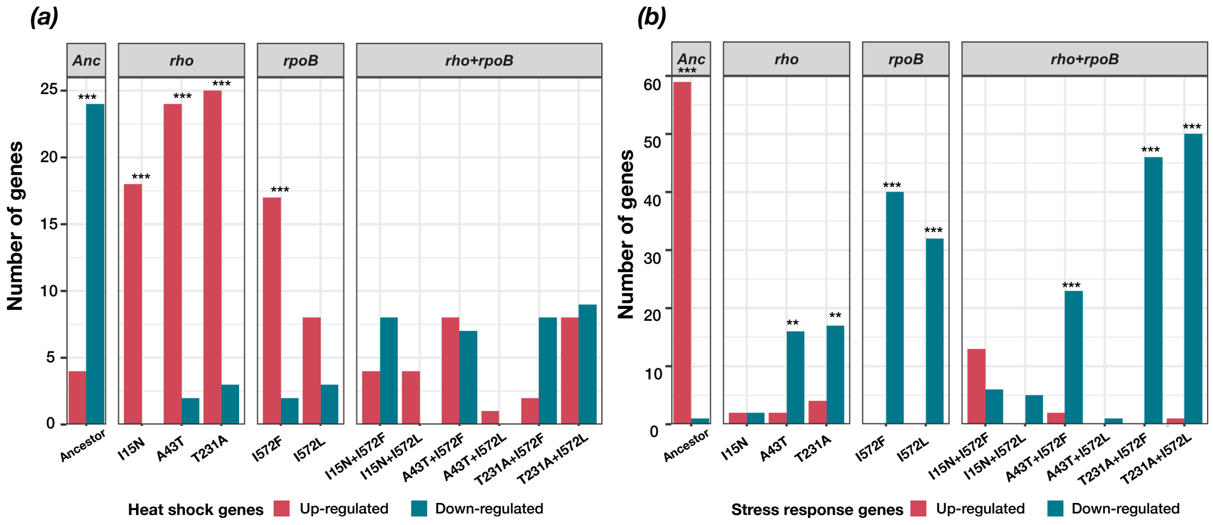

Figure S5: Changes in expression of heat shock induced and general stress response genes. Bar plots showing the number of ***(a)*** heat shock and ***(b)*** general stress response genes that are differentially expressed in each *rho+rpoB* double mutant and each *rho* and *rpoB* single mutant relative to the Ancestor at 42.2°C. Asterisks represent statistical difference (binomial test) between the number of up and down regulated genes. *** $P < 0.001$, ** $P < 0.01$ and, * $P < 0.05$. Heat shock induced genes were taken from Riehle et al. (2003), Nanoka et al. (2006), Gunasekera et al. (2008). General stress response list of genes was taken from Weber et al. (2005). See Literature Cited of the main text for the complete references.

(a)

	I15N + I572F	I15N + I572L	A43T + I572F	A43T + I572L	T231A + I572F	T231A + I572L
lamB	11.19	4.75	13.12	7.77	11.97	6.46
malE	10.00	4.30	12.86	7.75	10.55	6.37
malF	9.51	4.15	11.90	6.86	10.21	5.85
malM	11.34	4.54	11.74	6.31	10.42	4.87
malK	8.00	3.71	11.34	7.57	9.55	5.71
malG	9.46	3.45	11.08	5.16	9.28	4.12
malQ	8.25	2.35	9.69	4.84	7.49	4.16
tdcB	6.34	4.43	8.66	6.39	7.47	3.63
malS	6.76	2.73	8.61	4.83	5.64	3.12
ydeN	5.31	2.78	7.86	4.99	4.51	3.12
uidC	4.71	2.32	7.36	4.40	6.23	3.02
xylF	5.80	3.92	7.16	5.55	5.94	3.44
ydcV	6.33	2.67	7.07	5.10	4.67	2.44
ydcS	6.28	3.22	6.78	5.77	4.11	3.07
ydcT	6.13	2.75	6.56	5.22	4.95	3.13
glcC	4.38	3.46	6.52	4.58	4.32	3.47
ygeV	5.01	2.62	6.45	4.68	4.77	3.23
astC	4.74	2.86	6.31	4.49	3.69	2.01
aldB	5.42	2.51	5.89	4.48	3.81	2.44
ECB0351	4.06	2.70	5.75	4.02	4.02	2.57
fadB	3.90	2.76	5.69	3.69	3.79	2.40
tnaA	4.56	2.89	5.66	4.24	5.49	3.14
ydcU	5.30	2.31	5.52	4.48	3.86	2.21
ECB0351	4.12	2.75	5.44	3.81	4.44	2.83
yeiL	3.79	2.50	5.15	4.24	4.05	3.23
alsA	3.59	3.41	4.77	3.78	5.65	2.55
lldP	3.41	2.66	4.65	2.95	4.01	2.12
idnK	3.48	2.39	4.34	3.37	3.72	2.58
tdcA	3.39	3.11	4.12	3.86	3.79	3.27
eutP	3.39	2.80	3.95	3.39	3.87	2.14
ygfJ	3.53	3.40	3.85	5.25	4.23	2.69
tnaB	3.22	3.23	3.64	3.71	5.39	2.71

(b)

	I15N	A43T	T231A	I572F	I572L	I15N + I572F	I15N + I572L	A43T + I572F	A43T + I572L	T231A + I572F	T231A + I572L
lamB	-4.22	-5.79	-6.97	-4.24	-0.81	2.73	-0.28	3.09	1.17	0.75	-1.32
malE	-3.59	-5.63	-5.89	-4.14	-1.21	2.27	-0.49	3.09	0.92	0.51	-0.73
malF	-3.89	-5.18	-5.70	-4.06	-1.14	1.56	-0.88	2.66	0.53	0.46	-0.99
malM	-4.32	-4.45	-5.29	-4.42	-0.75	2.61	-0.53	2.88	1.12	0.71	-1.17
malK	-2.82	-5.66	-5.67	-3.14	-0.73	2.03	0.15	2.54	1.18	0.74	-0.70
malG	-3.25	-3.88	-4.52	-3.55	-0.09	2.67	0.11	3.66	1.19	1.21	-0.49
malQ	-3.23	-3.55	-3.79	-2.87	-0.33	2.15	-1.21	3.27	0.96	0.83	0.04
tdcB	-1.81	-4.78	-3.97	-5.53	-3.76	-1.00	-1.14	-1.65	-2.14	-2.04	-4.10
malS	-2.53	-3.38	-3.47	-2.36	-0.90	1.87	-0.71	2.87	0.55	-0.19	-1.26
ydeN	-1.30	-3.64	-3.25	-4.41	-2.18	-0.40	-0.70	-0.19	-0.83	-3.16	-2.31
uidC	-2.29	-4.37	-4.66	-5.14	-2.63	-2.72	-2.60	-2.14	-2.60	-3.56	-4.27
xylF	-2.00	-3.67	-3.56	-4.72	-3.86	-0.92	-1.93	-1.24	-1.97	-2.35	-3.98
ydcV	-0.68	-3.03	-3.07	-4.32	-2.80	1.33	-0.81	-0.27	-0.73	-2.72	-3.43
ydcS	-0.73	-2.59	-2.86	-3.65	-3.51	1.90	-1.02	0.54	-0.34	-2.40	-3.31
ydcT	-0.59	-2.29	-2.99	-3.73	-3.07	1.82	-0.90	0.53	-0.14	-1.77	-2.93
glcC	-1.62	-2.19	-2.79	-2.93	-2.91	-0.17	-1.07	1.40	-0.52	-1.40	-2.24
ygeV	-1.11	-2.48	-2.89	-3.83	-2.94	0.06	-1.43	0.13	-0.74	-1.96	-2.60
astC	-0.50	-2.14	-2.67	-3.59	-3.15	0.65	-0.80	0.57	-0.81	-2.58	-3.82
aldB	-0.37	-2.16	-2.44	-3.45	-2.89	1.60	-0.76	0.28	-0.58	-2.08	-2.89
ECB0351	-0.94	-2.30	-2.21	-3.45	-2.54	-0.34	-0.78	0.00	-0.82	-1.63	-2.18
fadB	-1.01	-1.74	-2.94	-2.97	-2.35	-0.08	-0.60	0.98	-0.40	-2.12	-2.88
tnaA	-1.39	-3.21	-3.32	-5.38	-3.59	-2.21	-2.09	-2.93	-2.57	-3.22	-3.77
ydcU	-0.49	-2.08	-2.34	-3.11	-2.41	1.70	-0.59	0.34	-0.01	-1.59	-2.54
ECB0351	-1.06	-2.25	-2.30	-3.90	-2.65	-0.83	-0.95	-0.71	-1.09	-1.76	-2.12
yeiL	-1.40	-2.58	-2.49	-2.66	-2.42	-0.28	-1.33	-0.09	-0.76	-1.11	-1.69
alsA	-2.01	-3.23	-3.48	-3.64	-2.72	-2.06	-1.31	-2.10	-2.16	-1.47	-3.65
lldP	-1.34	-1.87	-2.82	-2.82	-2.24	-0.75	-0.91	-0.04	-1.15	-1.63	-2.94
idnK	-0.91	-1.68	-1.73	-2.26	-2.17	0.31	-0.69	0.40	-0.48	-0.27	-1.32
tdcA	-1.37	-2.50	-2.36	-3.52	-3.61	-1.50	-1.87	-1.90	-2.25	-2.09	-2.70
eutP	-1.37	-2.45	-1.76	-2.92	-2.46	-0.90	-1.03	-1.43	-1.53	-0.81	-2.08
ygfJ	-1.20	-2.64	-2.51	-3.45	-4.17	-1.13	-1.98	-2.24	-1.55	-1.73	-3.99
tnaB	-1.34	-2.84	-3.24	-3.19	-2.85	-1.31	-0.96	-2.39	-1.98	-1.05	-3.38

(c)

	I15N + I572F	I15N + I572L	A43T + I572F	A43T + I572L	T231A + I572F	T231A + I572L
rpsR	-4.30	-2.41	-3.40	-2.49	-3.45	-2.24
rplB	-4.34	-2.80	-3.42	-2.66	-3.77	-2.27
rpsF	-4.34	-3.02	-3.45	-2.92	-3.36	-2.70
rpsJ	-4.49	-3.06	-3.54	-2.62	-3.65	-2.21
rpsI	-4.21	-2.89	-3.61	-2.82	-3.83	-2.53
rplK	-4.37	-2.72	-3.62	-2.61	-3.62	-2.27
rplD	-4.73	-3.18	-3.66	-2.89	-3.90	-2.52
rplA	-4.44	-2.74	-3.69	-2.60	-3.77	-2.42
rplC	-4.82	-3.34	-3.88	-2.93	-4.07	-2.63
yeeF	-5.10	-3.44	-3.95	-2.62	-3.75	-2.19
rplI	-4.95	-2.91	-3.96	-2.89	-4.01	-2.57
metR	-4.45	-2.49	-4.95	-3.61	-4.35	-3.00
metN	-4.74	-3.18	-4.97	-3.85	-4.21	-2.67
metF	-5.13	-2.90	-5.21	-3.55	-4.32	-2.39
ydjO	-5.39	-3.62	-5.59	-4.43	-4.01	-2.17
bioC	-5.18	-2.40	-5.93	-3.29	-5.45	-3.56
metE	-5.73	-3.07	-6.31	-4.20	-6.30	-4.23
bioF	-5.67	-2.65	-6.43	-3.57	-5.87	-3.81
metA	-5.64	-3.11	-6.56	-4.61	-6.06	-3.70
ydjN	-6.78	-3.92	-7.18	-4.49	-6.09	-3.57
metK	-6.54	-3.82	-7.44	-4.97	-6.48	-4.05
yqqD	-6.31	-3.32	-8.32	-4.84	-5.64	-3.36

Epistatic score (ε_{exp})

< -6 | < -4 | < -2 | > 2 | > 4 | > 6 | > 9

(d)

	I15N	A43T	T231A	I572F	I572L	I15N + I572F	I15N + I572L	A43T + I572F	A43T + I572L	T231A + I572F	T231A + I572L
rpsR	1.98	3.03	3.28	3.56	2.88	1.24	2.45	3.19	3.42	3.39	3.92
rplB	2.13	3.41	3.59	3.68	2.98	1.47	2.32	3.67	3.73	3.50	4.30
rpsF	1.90	2.84	3.11	3.70	3.42	1.26	2.29	3.09	3.34	3.44	3.83
rpsJ	2.16	3.13	3.29	3.69	3.22	1.36	2.32	3.28	3.73	3.33	4.30
rpsI	2.70	3.46	3.70	3.68	2.96	2.17	2.77	3.53	3.60	3.55	4.13
rplK	2.36	3.17	3.46	3.29	2.79	1.28	2.43	2.84	3.35	3.13	3.98
rplD	2.61	3.69	3.89	3.94	3.35	1.82	2.78	3.97	4.15	3.93	4.72
rplA	2.38	3.22	3.54	3.38	2.83	1.33	2.48	2.91	3.45	3.15	3.95
rplC	2.49	3.54	3.77	4.02	3.43	1.68	2.58	3.68	4.04	3.72	4.57
yeeF	2.88	2.80	3.25	2.89	3.39	0.67	2.83	1.74	3.57	2.39	4.45
rplI	2.78	3.75	3.95	4.20	3.30	2.03	3.17	3.99	4.15	4.14	4.68
metR	2.36	3.02	2.82	0.99	1.11	-1.11	0.98	-0.94	0.52	-0.54	0.94
metN	2.22	2.72	2.91	1.72	1.90	-0.79	0.93	-0.53	0.77	0.42	2.14
metF	2.88	3.12	3.18	1.32	1.33	-0.93	1.31	-0.77	0.90	0.18	2.11
ydjO	2.31	2.52	2.68	2.47	2.83	-0.61	1.52	-0.61	0.92	1.13	3.34
bioC	1.11	2.31	2.43	2.60	2.21	-1.47	0.92	-1.03	1.23	-0.42	1.07
metE	1.75	4.05	4.38	3.53	3.23	-0.45	1.91	1.27	3.07	1.61	3.38
bioF	1.51	2.64	2.75	2.70	2.25	-1.47	1.10	-1.09	1.32	-0.42	1.19
metA	3.33	4.35	4.18	2.20	2.13	-0.11	2.35	-0.01	1.87	0.32	2.61
ydjN	3.31	4.22	4.47	4.50	3.47	1.03	2.85	1.54	3.19	2.88	4.36
metK	2.73	3.81	3.94	2.68	2.24	-1.14	1.14	-0.94	1.09	0.14	2.13
yqqD	2.58	3.60	3.62	2.51	1.98	-1.22	1.24	-2.21	0.74	0.49	2.25

Mutant/Anc42 (log_2 Fold Change)

< -5 | < -4 | < -3 | < -2 | < -1 | < 0 | > 0 | > 1 | > 2 | > 3 | > 4

Figure S6. Gene expression epistasis. Highly significant epistatic events shared across all six *rho+rpoB* mutants. Significant epistatic events are associated to those genes that are highly differentially expressed in any of the *rho*, *rpoB* or *rho+rpoB* mutants ($q < 0.001$ and log_2 fold change > 2 or < -2) and that have a Z-score of > 2σ or < - 2σ (see Material and Methods for more details). Panels ***(a)*** and ***(c)*** depict those genes with significant positive and negative epistatic interactions respectively. Magenta and green palettes indicate different levels of positive and negative epistasis score (ε_{exp}) respectively. Panels ***(b)*** and ***(d)*** present the levels of expression (log_2 fold change) of each *rho*, *rpoB,* and *rho+rpoB* mutant.

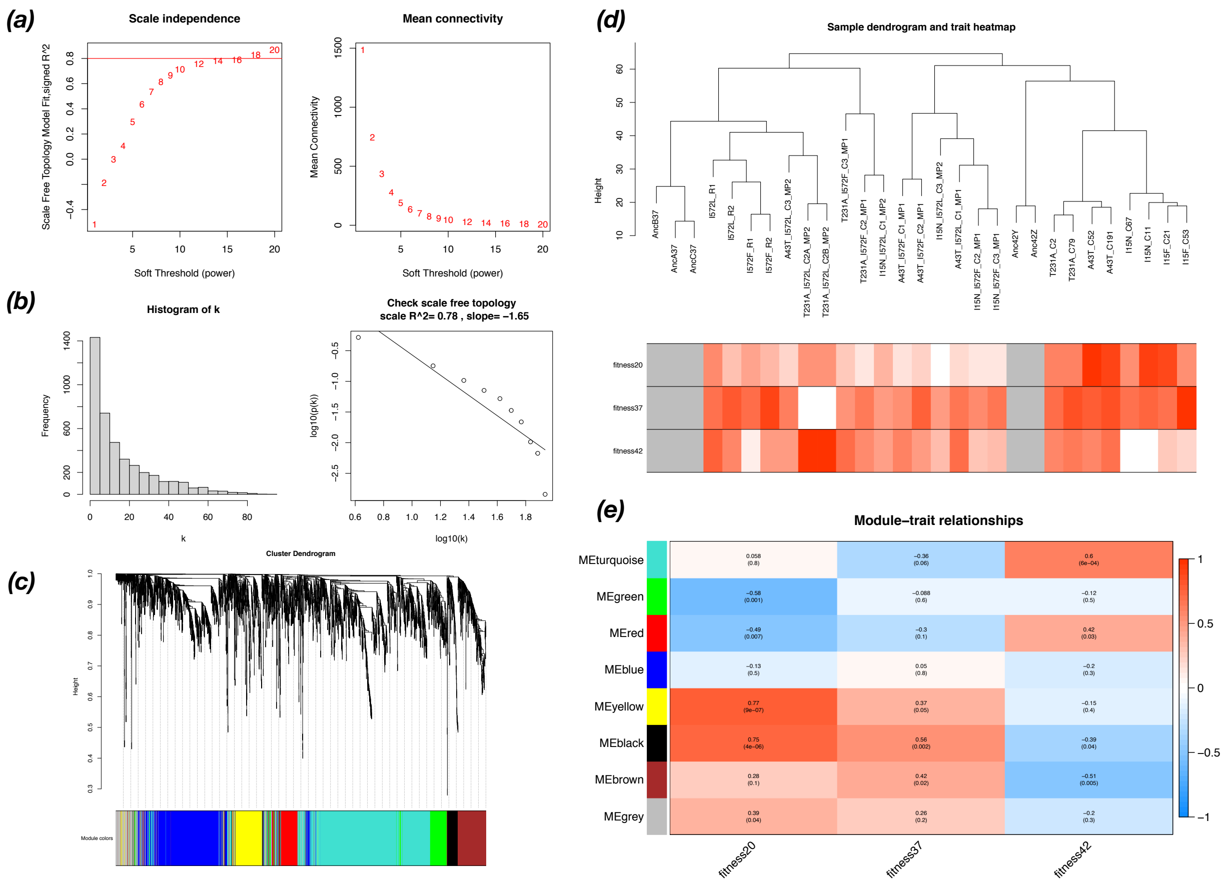

Figure S7: Co-expression network construction and detection of gene modules

Panels ***(a)*** and ***(b)*** represent the network topology analysis to select the soft-thresholding power. Our data does not fit the assumption of the scale-free topology approximation because the scale-free topology fit index does not reach values above 0.8 (we obtained 0.78). This lack of scale-free topology fit is caused by biological heterogeneity (marked differences among genotypes) and does not invalidate our data. As suggested by the authors of WGCNA, we chose 16 as soft-thresholding power in order to obtain a conservative network considering that we have 29 samples and run a signed network ***(c)*** Hierarchical clustering dendogram of genes with their assigned co-expression module underneath. Non-assigned genes are colored in gray. ***(d)*** Visualization of the relative fitness at different temperatures related to the sample dendogram. White refers to low values, red to high values, and gray to missing data. ***(e)*** Module-trait associations. Heatmap of the correlation between module eigengen expression and sample traits. Each column corresponds to a measured trait (Relative fitness at 20.0°C, 37.0°C and, 42.2°C) and each row to a different module. Within each cell the top number indicates the correlation coefficient and the bottom, the p-value. Red colors refer to positive correlation and blue to negative correlation.

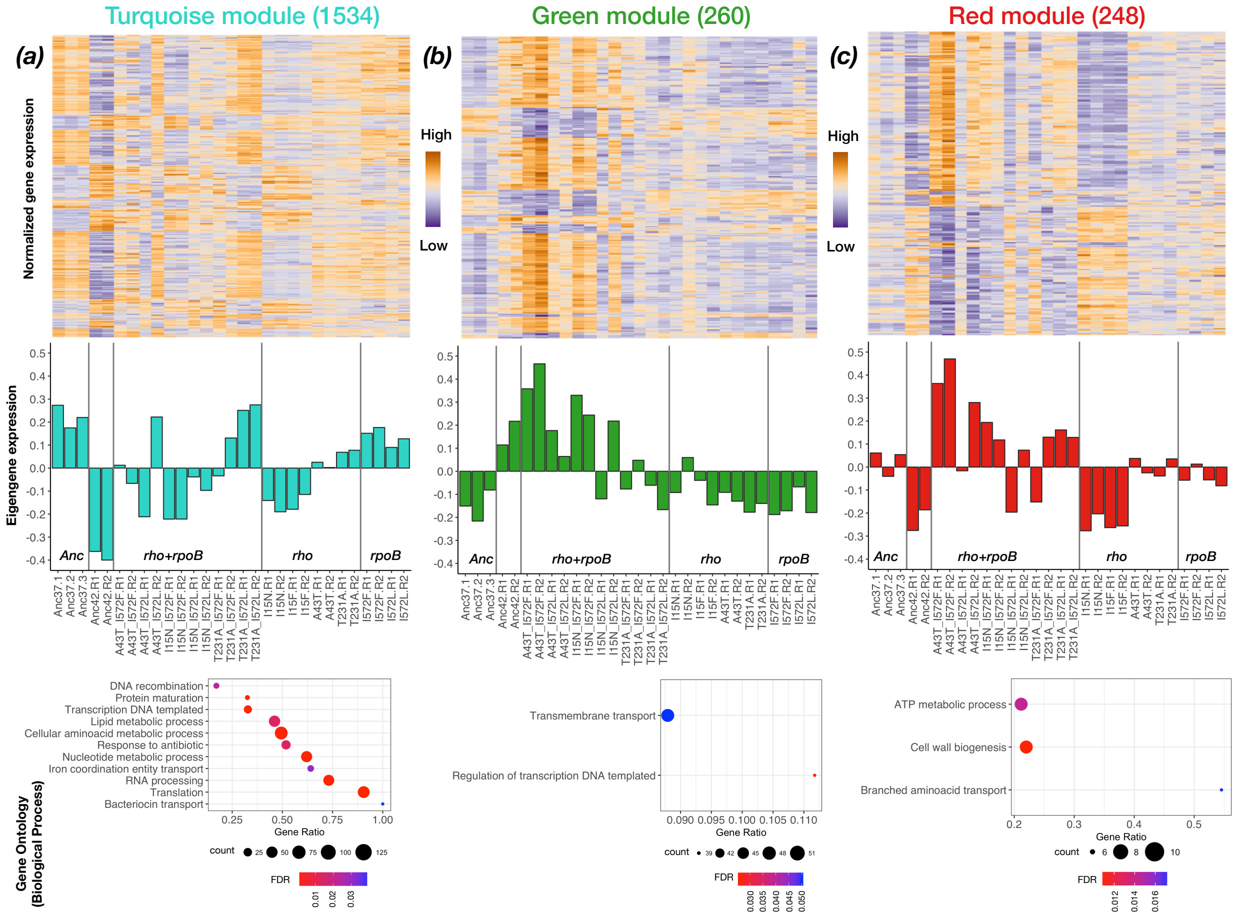

Turquoise module (1534)
Green module (260)
Red module (248)
(a)
(b)
(c)
Normalized gene expression
High
Low
Eigengene expression
Anc
rho+rpoB
rho
rpoB
Gene Ontology (Biological Process)
DNA recombination
Protein maturation
Transcription DNA templated
Lipid metabolic process
Cellular aminoacid metabolic process
Response to antibiotic
Nucleotide metabolic process
Iron coordination entity transport
RNA processing
Translation
Bacteriocin transport
Transmembrane transport
Regulation of transcription DNA templated
ATP metabolic process
Cell wall biogenesis
Branched aminoacid transport
Gene Ratio
count
FDR

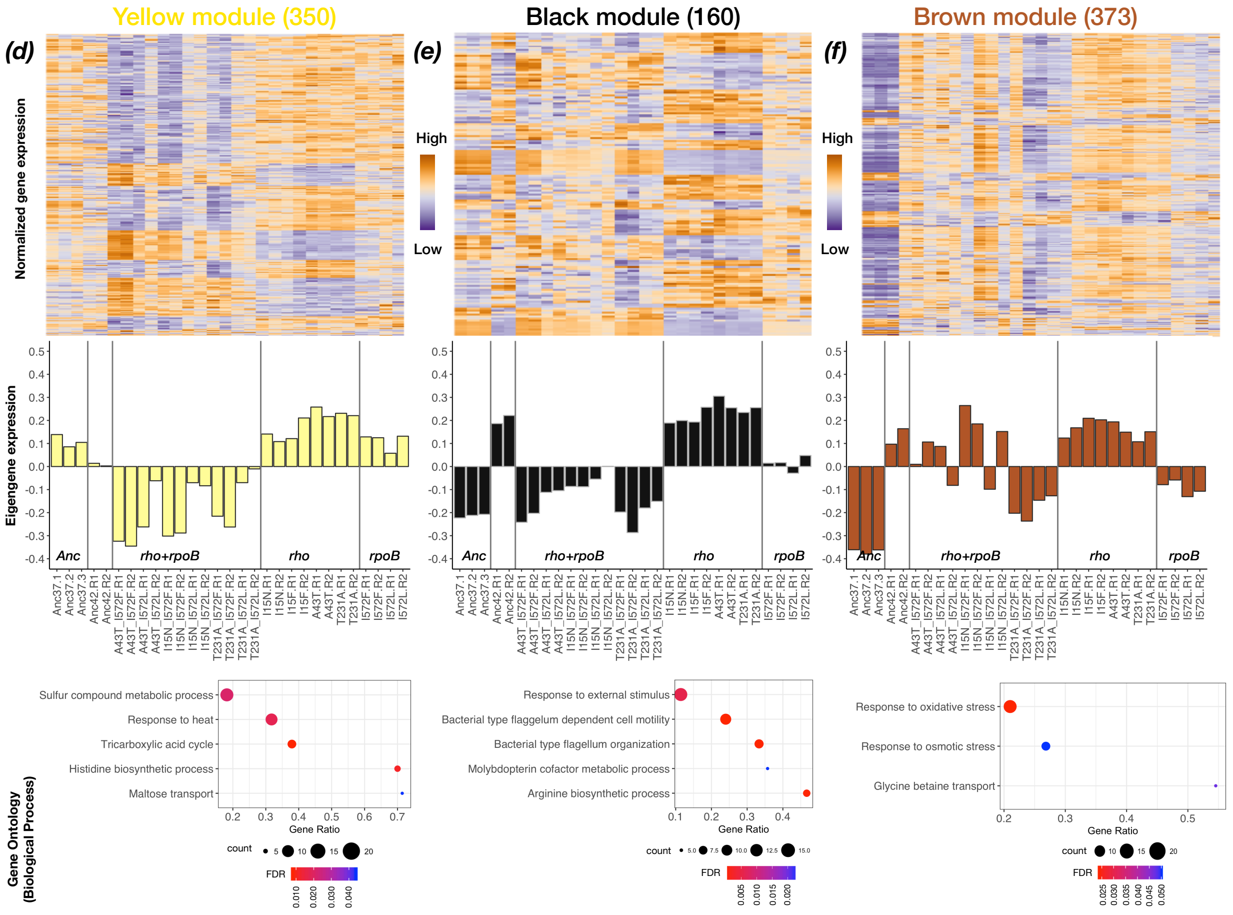

Figure S8. Previous figure and this represent the characterization of gene modules significantly associated to relative fitness at different temperatures per module (***a-e***) Heatmaps (top panels) of the normalized gene expression (variance stabilizing transformation, rows refer to genes and columns to samples) of all genes per module where purple and orange colors denote high and low expression levels respectively. The number of genes per module is in parenthesis. Barplots (middle panels) depict the expression levels (y-axis) of the corresponding eigengen per sample (x-axis). Samples with high expression levels (orange), have positive eigengene expression values while samples with low expression levels (purple) have negative eigengene expression values. Dotplots (bottom panels) show the significant enriched biological processes per module. Count is the number of genes belonging to each GO term detected per module. Gene ratio is obtained by dividing count by the total number of genes associated with that particular GO term in the *E. coli* genome. False Discovery rate (FDR)-adjusted *P value* refers to the significance associated to the Gene Ontology enrichment analysis obtained by a Binomial test and False Discovery Rate correction for multiple testing.

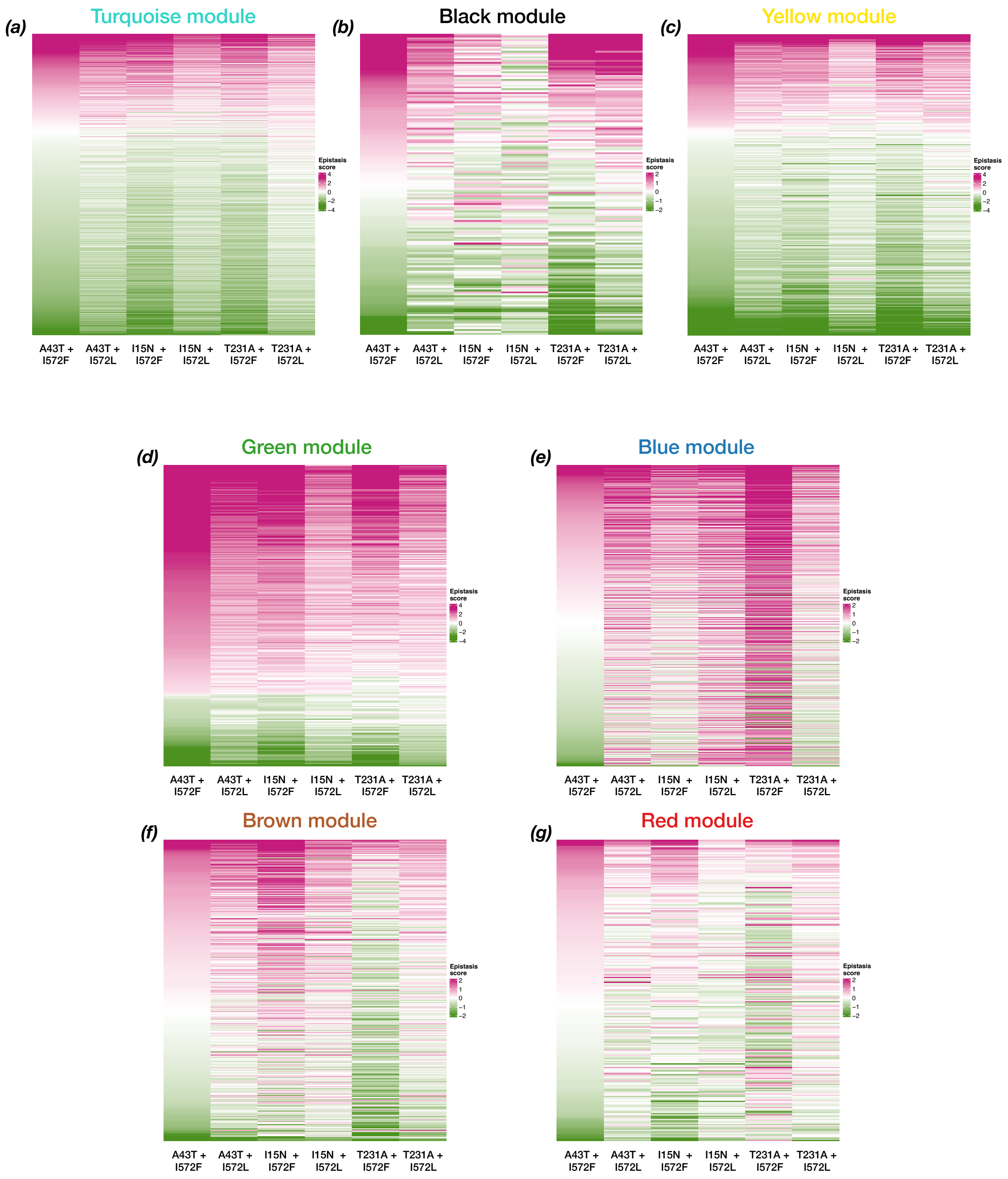

Figure S9. Epistasis scores of co-expression gene modules

Heatmap showing the negative (ε_{exp} green shades) or positive (ε_{exp} pink shades) epistasis scores of all genes belonging to ***a***) turquoise, ***b***) black, ***c***) yellow, ***d***) green, ***e***) blue, ***f***) brown and, ***g***) red co-expression modules.

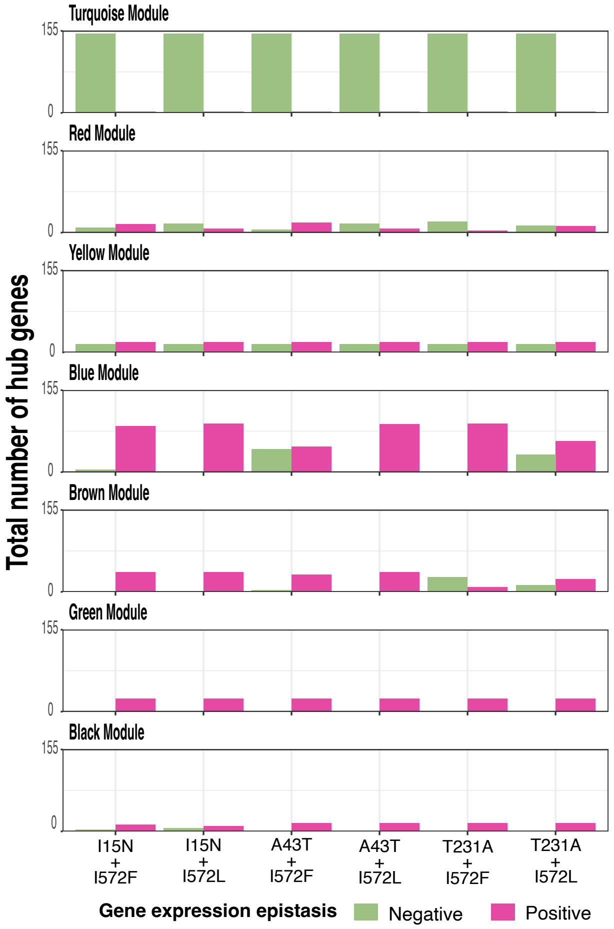

Figure S10. The number of hub genes within each module with either positive (exp>0) or negative gene expression epistasis (exp<0) scores for each of the six *rho+rpoB* mutants.

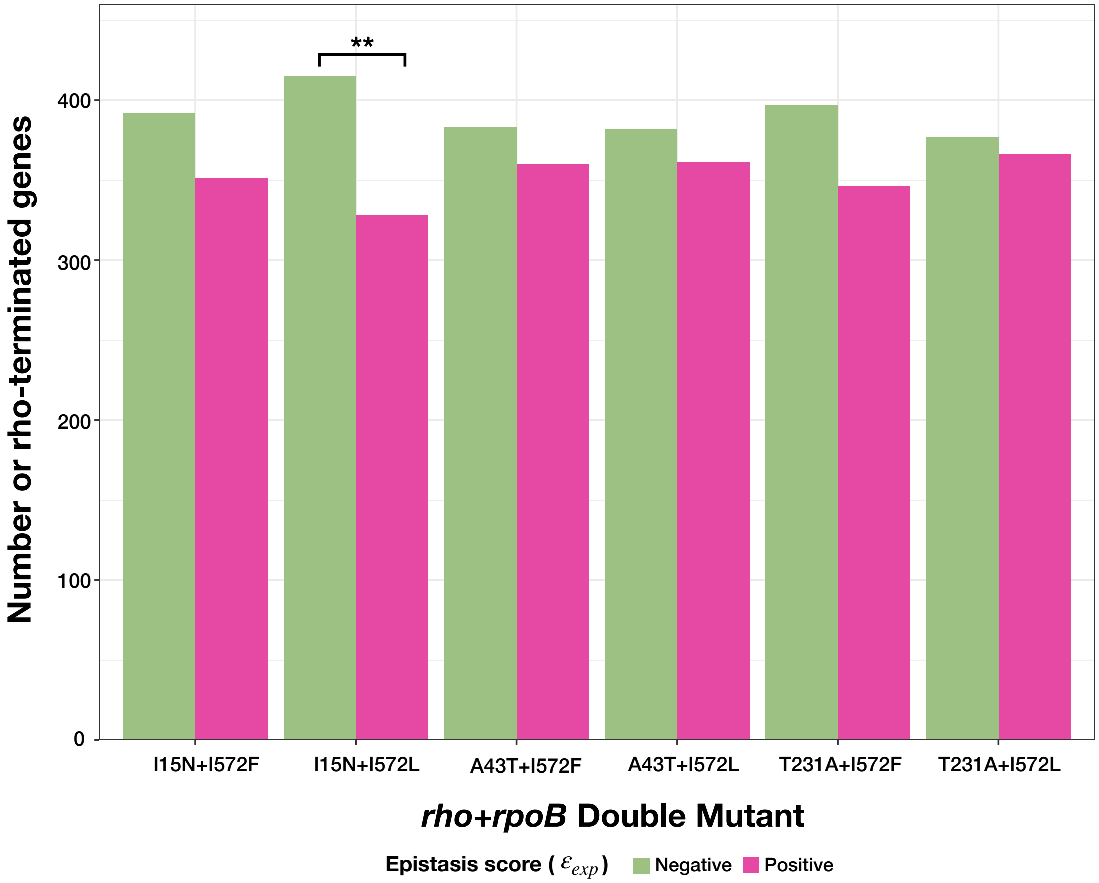

Figure S11. Number of rho-terminated genes with negative ($\varepsilon_{exp} < 0$) or positive epistasis ($\varepsilon_{exp} > 0$) scores per each single *rho+rpoB* Double mutant. Asterisks represent statistical difference (binomial test) between the number of genes under positive and negative epistasis. ** $P < 0.01$

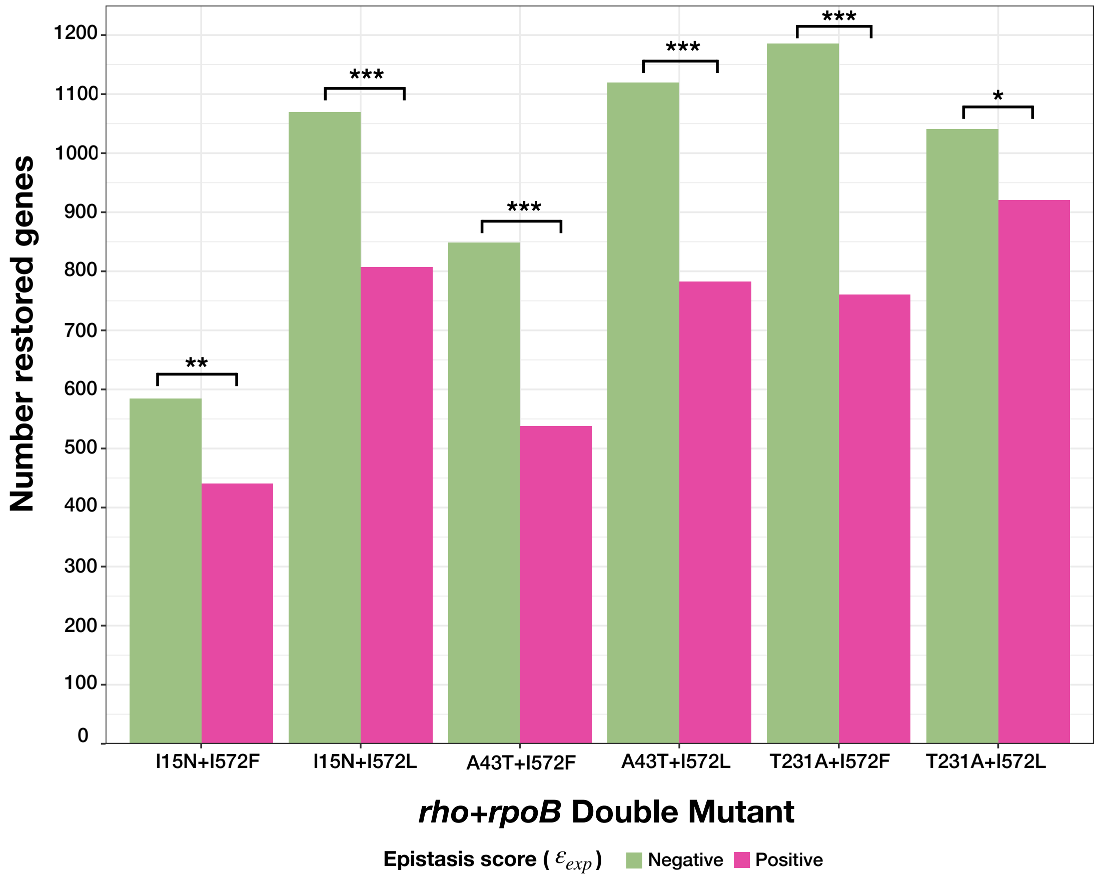

Figure S12. Number of restored genes with negative ($\varepsilon_{exp} < 0$) or positive epistasis ($\varepsilon_{exp} > 0$) scores per each single *rho+rpoB* Double mutant. Asterisks represent statistical difference (binomial test) between the number of genes under positive and negative epistasis. *** *P* < 0.001, ** *P* < 0.01
