# Supplementary material for: Patterns of Fitness and Gene Expression Epistasis Generated by Beneficial Mutations in the rho and rpoB Genes of Escherichia coli during High-Temperature Adaptation
Source: Mol Biol Evol. 2024 Sep 5;41(9):msae187. doi: 10.1093/molbev/msae187 (PMC11414761; doi:10.1093/molbev/msae187)
Supplement: msae187_Supplementary_Data [file msae187_supplementary_data.zip › MBE_SuppTables_082624.pdf]

Table S1. Mean relative fitness ($\overline{w}_r$) estimates of *rho* and *rpoB* single mutants at 42.2℃, 37.0℃ and 20.0℃.

**Temperature**	42.2℃			37.7℃			20.0℃		
**Mutant** [a]	***w*r (± 95% CI)**	**N**[b]	***P***[c]	***w*r (± 95% CI)**	**N**[b]	***P***[b]	**wr (± 95% CI)**	**N**[a]	***P***[b]
rhoA43T	1.237 (0.072)	9	<0.0001*	0.987 (0.052)	6	0.5550	1.015 (0.018)	9	0.0666
rhoT231A	1.266 (0.080)	9	<0.0001*	0.979 (0.049)	6	0.3166	0.956 (0.048)	9	0.0500
rhoI15F	1.008 (0.089)	9	0.83798	0.972 (0.050)	6	0.1864	0.988 (0.028)	9	0.3565
rhoI15N	0.987 (0.110)	8	0.79672	0.962 (0.019)	6	0.0005*	0.949 (0.017)	8	<0.0001*
rpoBI572F	1.154 (0.049)	6	<0.0001*	0.960 (0.037)	6	0.0230	0.921 (0.023)	6	<0.0001*
rpoBI572L	1.229 (0.104)	6	0.0002*	0.964 (0.030)	6	0.0136	0.940 (0.027)	6	0.0002*

[a] Mutant data for *rho* and *rpoB* mutations taken from Gonzalez-Gonzalez et al. (2017) and Rodriguez-Verdugo et al. (2014)

[b]Total number of replicates

[c]*P* < 0.05 rejects the null hypothesis $\overline{w}_r = 1.0$, suggesting that the mutation is beneficial if $\overline{w}_r > 1.0$ or detrimental if $\overline{w}_r < 1.0$ relative to the ancestor. Asterisks denote significant p-value at *P* < 0.05 after Bonferroni correction.

Table S2. Mean relative fitness ($\overline{w}_r$) estimates of *rho*+r*poB* double mutants at 42.2°C, 37.0°C and 20.0°C.

**Temp.**	42.2°C			37.0°C			20.0°C		
**Mutant**	**w_r (± 95% CI)[a]**	**N[a]**	***P*[b]**	**w_r (± 95% CI)[a]**	**N[a]**	***P*[b]**	**w_r (± 95% CI)[a]**	**N[a]**	***P*[b]**
***rho**A*43T**+ rpoB**I572F	1.196 (0.048)	12	<0.0001*	0.916 (0.015)	10	<0.0001*	0.907 (0.023)	12	<0.0001*
rhoA43T**+ *rpoB***I572L	1.252 (0.108)	9	<0.0001*	0.935 (0.018)	9	<0.0001*	0.908 (0.029)	9	<0.0001*
rhoT231A**+ *rpoB***I572F	1.220 (0.052)	12	<0.0001*	0.898 (0.019)	11	<0.0001*	0.878 (0.033)	10	<0.0001*
rhoT231A**+ *rpoB***I572L	1.376 (0.100)	12	<0.0001*	0.929 (0.022)	11	<0.0001*	0.937 (0.020)	12	<0.0001*
rhoI15F**+ *rpoB***I572F	1.127 (0.044)	10	<0.0001*	0.903 (0.028)	12	<0.0001*	0.868 (0.037)	12	<0.0001*
rhoI15*F*+ *rpoB***I572L	1.237 (0.071)	7	<0.0001*	0.915 (0.025)	9	<0.0001*	0.937 (0.038)	9	<0.0015*
rhoI15N**+ *rpoB***I572F	1.077 (0.067)	9	0.01754	0.892 (0.027)	11	<0.0001*	0.892 (0.031)	12	<0.0001*
rho*I**15N**+ *rpoBI572L	1.169 (0.110)	7	0.00269*	0.892 (0.018)	9	<0.0001*	0.880 (0.032)	7	<0.0001*

[a]Total number of replicates

[b]*P* < 0.05 rejects the null hypothesis $\overline{w}_r = 1.0$, suggesting that the mutation is beneficial if $\overline{w}_r > 1.0$ or detrimental if $\overline{w}_r < 1.0$ relative to the ancestor. * denotes *P* value <0.05 after Bonferroni correction.

Table S3. Epistatic deviation ε_{wr} of *rho*+*poB* double mutants at 42.2℃ calculated using relative fitness ($\bar{w}_r$) estimates

Mutant	$\bar{w}_{rxy}$ [a] (Var)	$\bar{w}_{rx}\bar{w}_{ry}$ [b] (Var)	ε [c] (Var)	*P* [d]
rho*A*43T+***rpoB***I572F	1.196 (0.0050)	1.429 (0.0131)	-0.2325 (0.0182)	**<0.000**
rhoA43T+***rpoB***I572L	1.252 (0.0175)	1.520 (0.0243)	-0.2680 (0.0418)	**0.0043**
rhoT231A+***rpoB***I572F	1.220 (0.0061)	1.462 (0.0157)	-0.2430 (0.0218)	**0.0001**
rhoT231A+***rpoB***I572L	1.376 (0.0229)	1.556 (0.0276)	-0.1802 (0.0505)	**0.0180**
rhoI15F+***rpoB***I572F	1.127 (0.0035)	1.164 (0.0180)	-0.037 (0.0215)	0.4431
rhoI15*F*+***rpoB***I572L	1.237 (0.0050)	1.239 (0.0267)	-0.001 (0.0318)	0.9866
rhoI15N ***+ rpoB***I572F	1.077 (0.0068)	1.141 (0.0219)	-0.0635 (0.02874)	0.2938
rho*I**15N **+ *rpoBI572L	1.169 (0.0121)	1.2137 (0.0309)	-0.0446 (0.0430)	0.5898

[a] Mean of the observed relative fitness and its variance
[b] Mean of the expected relative fitness and its variance
[c] Absolute epistatic deviation and its variance
[d] Two-tailed *P* < 0.05 rejects the null hypothesis ε_{wr} *= 0,* ε_{wr} *> 0* and ε_{wr} *< 0* suggest positive and negative epistatic interactions between *rho* and *rpoB* mutations respectively.

Table S4. Epistatic deviation ε_{wr} of *rho*+*poB* double mutants at 37.0℃ calculated using relative fitness ($\overline{w}_r$) estimates

Mutant	$\overline{\boldsymbol{w}}_{\boldsymbol{rxy}}$ [a] (Var)	$\overline{\boldsymbol{w}}_{\boldsymbol{rx}}\overline{\boldsymbol{w}}_{\boldsymbol{ry}}$ [b] (Var)	$\boldsymbol{\varepsilon}$ [c] (Var)	*P* [d]
*rhoA*43T+*rpoB*I572F	0.916 (0.0004)	0.948 (0.0029)	-0.0319 (0.0033)	0.116
*rho*A43T+*rpo*BI572L	0.935 (0.0005)	0.952 (0.0026)	-0.0171 (0.0031)	0.383
*rho*T231A+*rpoB*I572F	0.898 (0.0007)	0.941 (0.0028)	-0.0430 (0.0034)	**0.036**
*rho*T231A+*rpoB*I572L	0.929 (0.0010)	0.945 (0.0024)	-0.0155 (0.0034)	0.401
*rho*I15F+*rpoB*I572F	0.903 (0.0017)	0.934 (0.0028)	-0.0304 (0.0045)	0.146
*rho*I15*F*+*rpoB*I572L	0.915 (0.0009)	0.937 (0.0024)	-0.0224 (0.0033)	0.281
*rho*I15N+*rpoB*I572F	0.892 (0.0015)	0.924 (0.0013)	-0.0318 (0.0027)	0.073
*rho*I15N+*rpoB*I572L	0.892 (0.0005)	0.928 (0.0009)	-0.0360 (0.0014)	**0.020**

[a] Mean of the observed relative fitness and its variance

[b] Mean of the expected relative fitness and its variance

[c] Absolute epistatic deviation and its variance

[d] Two-tailed $P < 0.05$ rejects the null hypothesis ε_{wr} *= 0,* ε_{wr} *> 0* and ε_{wr} *< 0* suggest positive and negative epistatic interactions between *rho* and *rpoB* mutations respectively.

Table S5. Epistatic deviation ε_{wr} of *rho*+*poB* double mutants at 20.0°C calculated using relative fitness ($\overline{w}_r$) estimates

Mutant	$\overline{\boldsymbol{w}}_{\boldsymbol{rxy}}$ [a] (Var)	$\overline{\boldsymbol{w}}_{\boldsymbol{rx}}\overline{\boldsymbol{w}}_{\boldsymbol{ry}}$ [b] (Var)	ε [c] (Var)	*P* [d]
rho*A*43T+***rpoB***I572F	0.907 (0.0012)	0.935 (0.0008)	-0.0283 (0.0020)	0.0508
rhoA43T+***rpo*B**I572L	0.9081 (0.0013)	0.9549 (0.0010)	-0.0468 (0.0023)	**0.0188**
rhoT231A+***rpoB***I572F	0.8781 (0.0020)	0.8805 (0.0033)	-0.0024 (0.0053)	0.9194
rhoT231A+***rpoB***I572L	0.9366 (0.0009)	0.8989 (0.0036)	0.0377 (0.0045)	0.0772
rhoI15F+***rpoB***I572F	0.8679 (0.0031)	0.9104 (0.0014)	-0.0425 (0.0045)	0.0509
rhoI15*F*+***rpoB***I572L	0.9373 (0.0022)	0.9295 (0.0016)	0.0078 (0.0037)	0.7137
rhoI15N ***+ rpoB***I572F	0.8925 (0.0022)	0.8741 (0.0006)	0.0184 (0.0029)	0.2600
rho*I**15N **+ *rpoBI572L	0.8806 (0.0011)	0.8924 (0.0008)	-0.0118 (0.0018)	0.4721

[a] Mean of the observed relative fitness and its variance

[b] Mean of the expected relative fitness and its variance

[c] Absolute epistatic deviation and its variance

[d] Two-tailed *P* < 0.05 rejects the null hypothesis $\varepsilon_{wr} = 0$, $\varepsilon_{wr} > 0$ and $\varepsilon_{wr} < 0$ suggest positive and negative epistatic interactions between *rho* and *rpoB* mutations respectively.

Table S6. Final yield of the rho+rpoB double mutants and rho and rpoB single mutants at 42.2°C.

		rho_*A43T*	rho_*I15F*	rho_*I15N*	rho_*T231A*	rpoB_*I572F*	rpoB_*I572L*
	Yield 42.2°C[a]	5.27×10^{6} (2.47×10^{5})	3.62×10^{6} (3.12×10^{5})	5.72×10^{6} (4.23×10^{5})	7.15×10^{6} (8.78×10^{4})	1.72×10^{7} (6.43×10^{5})	1.57×10^{7} (4.24×10^{5})
A43T+I572F	1.47×10^{7} (1.47×10^{5})	$\mathbf{3.24 \times 10^{-5}}$ [b]				0.055 [b]	
A43T+I572L	1.04×10^{7} (1.82×10^{5})	**0.000** [b]					**0.002** [b]
I15F+I572F	1.16×10^{7} (4.85×10^{5})		**0.000** [b]			**0.003** [b]	
I15F+I572L	1.07×10^{7} (7.52×10^{5})		**0.005** [b]				**0.009** [b]
I15N+I572F	1.09×10^{7} (7.69×10^{5})			**0.009** [b]		**0.004** [b]	
I15N+I572L	9.10×10^{6} (1.12×10^{6})			0.080 [b]			**0.017** [b]
T231A+I572F	2.06×10^{7} (1.88×10^{5})				$\mathbf{1.42 \times 10^{-5}}$ [b]	**0.025** [b]	
T231A+I572L	1.69×10^{7} (5.53×10^{5})				**0.003** [b]		0.161 [b]

[a] Final yield (cells/ml) ± standard error in parenthesis of rho and rpoB single mutants and rho and rpoB double mutants

[b] *P* values from two sample *t-test* testing the null hypothesis that values of the rho+rpoB double mutant and their respective rho or rpoB single mutant are the same. *P* <0.05 are in bold.

Table S7. Maximum growth rates of the *rho* and *rpoB* single mutants and the ancestor

Genotype	μ_{max}[a] 37°C	μ_{max}[a] 42.2°C
Ancestor	0.961 (0.006)	0.418 (0.033)
rho A43T	1.270 (0.043)	0.830 (0.184)
rho T231A	1.090 (0.007)	1.634 (0.407)
rho I15F	1.242 (0.042)	0.556 (0.046)
rho I15N	1.102 (0.007)	1.506 (0.330)
rpoB I572F	0.875 (0.085)	0.734 (0.021)
rpoB I572L	0.898 (0.090)	0.809 (0.092)
*rhoA*43T+*rpoB*I572F	0.943 (0.051)	0.590 (0.033)
*rho*A43T+*rpo*BI572L	1.024 (0.035)	0.998 (0.126)
*rho*T231A+*rpoB*I572F	0.894 (0.008)	0.734 (0.057)
*rho*T231A+*rpoB*I572L	0.942 (0.011)	0.742 (0.032)
*rho*I15F+*rpoB*I572F	0.940 (0.012)	0.745 (0.083)
*rho*I15*F*+*rpoB*I572L	0.911 (0.010)	0.631 (0.037)
*rho*I15N+*rpoB*I572F	0.960 (0.0015)	0.720 (0.051)
*rho*I15N+*rpoB*I572L	0.977 (0.0005)	0.712 (0.096)

[a] Maximum growth rate ± standard error in parenthesis.

Table S8: Gene Ontology (GO) enrichment analysis of the differentially expressed genes compared to the Ancestor at 42.2°C ($q < 0.001$) shared among all six rho+rpoB double mutants (2700 DEGs).

Biological Function	Gene	Name	*A43T* [1] + *I572F* / Anc42	*A43T* [1] + *I572L* / Anc42	*I15N* [1] + *I527F* / Anc42	*I15N* [1] + *I527L* / Anc42	*T231A* [1] + *I527F* / Anc42	*T231A* [1] + *I527L* / Anc42
**GO: 0071973** Bacterial-type flagellum-dependent cell motility	*flgC*	Flagellar basal-body rod protein FlgC	7.53	6.10	6.33	5.21	6.89	7.07
	flgD	Basal-body rod modification protein FlgD	7.29	5.81	6.09	4.75	6.67	6.61
	flgB	Flagellar basal body rod protein FlgB	7.23	5.59	5.86	4.43	6.40	6.46
	flgE	Flagellar hook protein FlgE	7.15	5.57	5.79	4.41	6.54	6.36
	flgG	Flagellar basal-body rod protein FlgG	7.12	5.36	5.55	4.21	6.61	6.19
	flgF	Flagellar basal-body rod protein FlgF	7.09	5.48	5.68	4.47	6.56	6.31
	flgH	Flagellar L-ring protein	6.62	4.67	5.02	3.60	6.36	5.68
	flgA	Flagella basal body P-ring formation protein FlgA	6.13	4.69	4.44	3.43	5.03	5.17
	flgI	Flagellar P-ring protein	6.05	4.15	4.44	3.28	5.80	5.19
	flgJ	Peptidoglycan hydrolase FlgJ	5.13	3.13	3.59	2.51	5.13	4.42
**GO: 0044780** Bacterial-type flagellum assembly	*flhA*	Flagellar biosynthesis protein FlhA	5.61	4.01	3.88	3.09	5.34	4.93
	flhB	Flagellar biosynthesis protein FlhB	5.82	4.26	4.06	3.29	5.48	5.53
	flgA	Flagella basal body P-ring formation protein FlgA	6.13	4.69	4.44	3.43	5.03	5.17
	flgJ	Putative peptidoglycan hydrolase FlgJ	5.13	3.13	3.59	2.51	5.13	4.42

	flgN	Flagellar biosynthesis protein FlgN	4.06	2.74	2.65	1.95	3.47	3.56
**GO: 0009260** Ribonucleotide biosynthetic process	*apt*	Adenine phosphoribosyltransferase	1.95	2.35	1.40	1.97	2.41	3.08
	carA	Carbamoyl phosphate synthetase subunit α	1.84	2.76	1.07	2.35	2.54	3.47
	carB	Carbamoyl phosphate synthetase subunit β	2.25	3.01	1.23	2.31	2.52	3.38
	pyrD	Dihydroorotate dehydrogenase, type 2	2.72	2.78	1.92	2.59	3.42	3.75
	purA	Adenylosuccinate synthetase	2.67	2.13	2.00	1.68	2.11	2.53
	purD	Phosphoribosylamine—glycine ligase	2.75	2.35	1.42	1.39	2.54	2.75
	purC	Phosphoribosylaminoimidazole-succinocarboxamide synthase	3.00	2.69	2.16	2.28	2.86	3.48
	purH	Bifunctional AICAR transformylase/IMP cyclohydrolase	2.97	2.80	1.87	2.09	3.09	3.42
	pyrE	Orotate phosphoribosyltransferase	3.83	4.61	2.83	4.10	4.65	5.43
	pyrB	Aspartate carbamoyltransferase catalytic subunit	4.45	5.86	3.80	5.46	4.62	6.62
	pyrF	Orotidine-5'-phosphate decarboxylase	1.79	2.76	0.91	2.48	2.51	3.67
	upp	Uracil phosphoribosyltransferase	2.47	2.40	1.81	2.06	2.48	3.08
**GO:0042255** Ribosome assembly	*rpsP*	30S ribosomal subunit protein S16	2.43	2.68	0.95	1.71	2.44	3.32
	rplS	50S ribosomal subunit protein L19	2.66	2.62	0.97	1.67	2.47	3.24
	rplK	50S ribosomal subunit protein L11	2.84	3.35	1.28	2.43	3.13	3.98
	rplB	50S ribosomal subunit protein L2	3.67	3.73	1.47	2.32	3.50	4.30
	rplE	50S ribosomal subunit protein L5	2.42	2.95	1.05	1.88	2.77	3.44
	rpsU	30S ribosomal subunit protein S21	2.55	2.15	2.05	2.33	2.93	3.43
	rplT	50S ribosomal subunit protein L20	2.97	2.75	1.71	1.90	2.91	3.34
	rplX	50S ribosomal subunit protein L24	2.62	2.88	1.22	1.95	2.73	3.49

	rplC	50S ribosomal subunit protein L3	3.68	4.04	1.68	2.58	3.72	4.57
	rpmG	50S ribosomal subunit protein L33	2.46	2.39	1.53	1.90	2.48	3.07
	rpsK	30S ribosomal subunit protein S11	2.71	2.66	0.86	1.69	2.80	3.33
	rpmB	50S ribosomal subunit protein L28	2.35	2.27	1.54	1.89	2.43	3.03
	rplA	50S ribosomal subunit protein L1	2.91	3.45	1.33	2.48	3.15	3.95
	rpsG	30S ribosomal subunit protein S7	2.49	2.82	1.03	1.81	2.46	3.23
	bipA	50S ribosomal subunit assembly factor BipA	2.62	2.41	1.47	1.72	3.08	3.16
	rplD	50S ribosomal subunit protein L4	3.97	4.15	1.82	2.78	3.93	4.72
**GO:0019388**	*galK*	Galactokinase	-1.51	-2.13	-2.35	-2.75	-2.85	-2.59
Galactose catabolic	*galT*	Galactose-1-phosphate uridylyltransferase	-1.97	-2.43	-2.80	-3.21	-2.93	-2.92
process	*galE*	UDP-glucose 4-epimerase	-1.67	-2.30	-2.45	-2.91	-2.64	-2.75

[1] Values refer to the $\log_2$ Fold Change between the *rho+rpoB* double mutants and the ancestor grown at 42.2°C

Table S9. Gene expression changes (log_2 Fold Change and *p*-adj values) of *rho* and RNA polymerase genes and sigma factors across *rho+rpoB* double mutants relative to the Ancestor at 42.2°C

Gene	Name	***A43T*** [1] + ***I572F*** / Anc42	***A43T*** [1] + ***I572L*** / Anc42	***I15N*** [1] + ***I527F*** / Anc42	***I15N*** [1] + ***I527L*** / Anc42	***T231A*** [1] + ***I527F*** / Anc42	***T231A*** [1] + ***I527L*** / Anc42
rho	Transcription termination factor	-0.37 (0.293)	0.47 (0.590)	-0.36 (0.097)	-0.04 (0.947)	0.27 (0.548)	0.68 (0.001)
rpoA	α subunit of the RNA polymerase	2.47 (4.28E-29)	2.43 (0.000)	0.60 (0.001)	1.40 (0.003)	2.51 (5.73E-13)	3.05 (1.21E-73)
rpoB	β subunit of the RNA polymerase	1.90 (4.87E-17)	1.52 (0.031)	0.94 (3.15E-07)	0.39 (0.427)	1.37 (0.000)	1.86 (1.49E-27)
rpoC	β' subunit of the RNA polymerase	2.15 (2.67E-19)	1.48 (0.036)	0.99 (3.45E-06)	0.42 (0.415)	1.49 (0.000)	1.86 (7.34E-23)
rpoZ	ω subunit of the RNA polymerase	1.08 (3.03E-06)	0.98 (0.156)	0.67 (0.000)	0.67 (0.122)	0.97 (0.008)	1.50 (2.96E-21)
rpoD	RNA polymerase σ70	-0.55 (0.018)	-0.22 (0.813)	-0.70 (0.000)	-0.81 (0.050)	-0.81 (0.030)	0.08 (0.694)
rpoE	RNA polymerase σE	1.82 (4.05E-07)	0.59 (0.408)	1.04 (1.17E-05)	-0.17 (0.803)	0.19 (0.696)	0.70 (0.008)
rpoN	RNA polymerase σ54	0.37 (0.142)	-0.07 (0.940)	-0.14 (0.524)	-0.48 (0.360)	-0.47 (0.208)	0.07 (0.731)

[1] Values refer to the log_2 Fold Change and the *p*-adj (in parenthesis) between the *rho+rpoB* double mutants and the ancestor grown at 42.2°C.

Table S10. Properties of the score distributions for gene expression epistasis

rho+rpoB mutant	ε_{exp} ± 95%CI[a]	t_{4175}[b]	*P*[c]	Skewness (γ_1)[d]	Kurtosis (γ_2)[e]	Significant events (%)[f]
*I15N+I572*F	0.015 ± 0.046	0.65	0.517	0.576	7.073	4.40
I15N+I572L	0.089 ± 0.032	5.33	**9.94×10^{-8}**	0.372	3.875	3.04
*A43T+I572*F	0.075 ± 0.052	2.83	**4.71×10^{-3}**	1.184	9.078	3.92
A43T+I572L	0.195 ± 0.039	9.64	**2.20×10^{-16}**	0.706	5.400	3.73
T231A+I572F	0.149 ± 0.052	5.57	**2.72×10^{-8}**	0.687	5.105	3.20
T231A+I572L	0.064 ± 0.028	4.44	**9.37×10^{-6}**	0.479	6.305	3.76

[a] Average epistasis score across ($\bar{\varepsilon}_{exp}$) the genome of each *rho+rpoB* mutant ± 95% confidence interval.

[b] *t*-value from *t*.test and 4176 degrees of freedom corresponding to the total number of genes in the genome minus 1.

[c] *t*.test, P < 0.05 rejects the null hypothesis ε_{exp}= 0, suggesting the epistasis score average is positive if ε_{exp} > 0 or negative if ε_{exp}< 0.

[d] If the coefficient of skewness $\gamma_1 = 0$, the epistatic scores are normally distributed (symmetrical). If $\gamma_1 > 0$, the epistatic score distribution is positively skewed and most of the epistatic score values are smaller than the mean and positioned on the left side of the distribution.

[e] Pearson's measure of kurtosis. If the coefficient of kurtosis $\gamma_2 = 3$, the data is normally distributed (mesokurtic). If $\gamma_2 > 3$, the distribution of the data is leptokurtic with more extreme outliers than a Gaussian distribution.

[f] Percentage of genes within the genome exhibiting significant epistasis scores (differentially expressed gene: $\log_2$ fold change < 2 or > -2 and *q* < 0.001 in at least one mutant plus a standard epistasis standard score Z-score < 2 or > -2).
